# Supplementary material for: Neutralisation of the Immunoglobulin-Cleaving Activity of Streptococcus equi Subspecies equi IdeE by Blood Sera from Ponies Vaccinated with a Multicomponent Protein Vaccine
Source: Vaccines (Basel). 2025 Oct 17;13(10):1061. doi: 10.3390/vaccines13101061 (PMC12568127; doi:10.3390/vaccines13101061)
Supplement: Supplementary file 1 [file vaccines-13-01061-s001.zip › Suppl_Fig_S3_original_blots.pdf]

Original blot of Figure 2

The red box indicates the cropped area in Fig 2

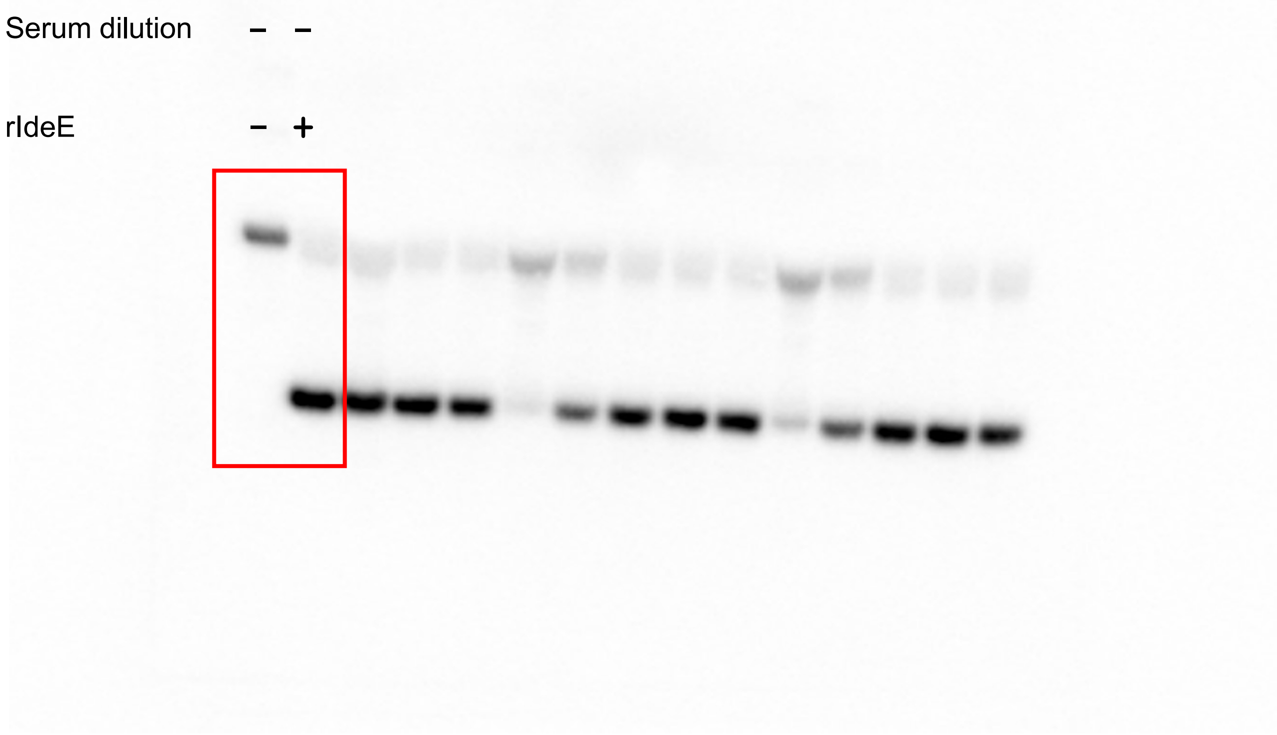

Titration of rIdeE

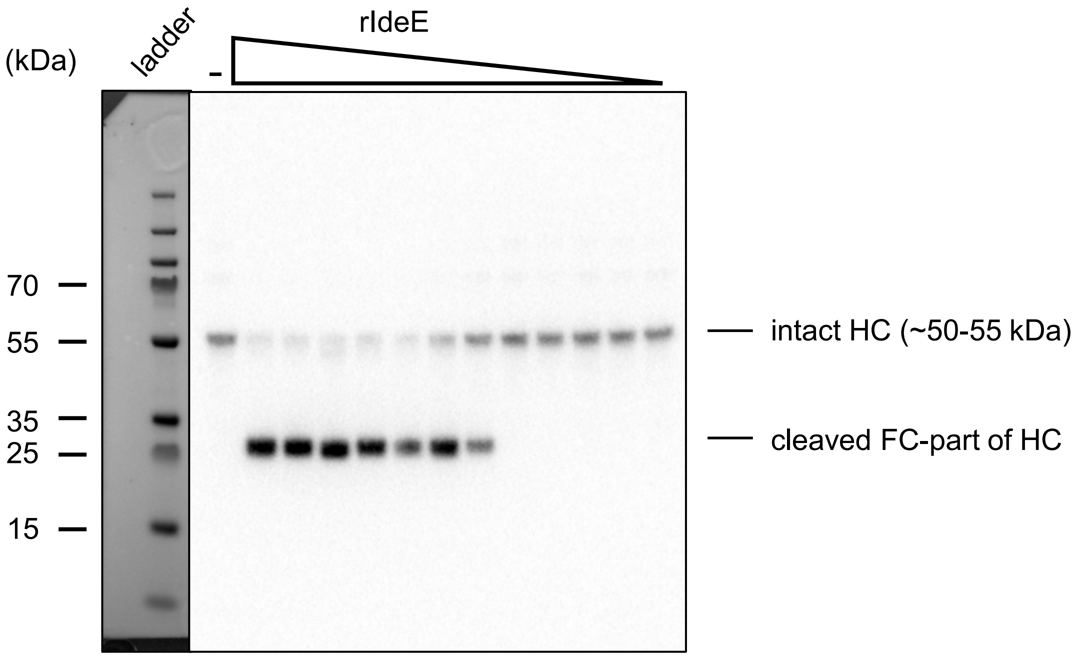

Exp I group 1

Pony 0292

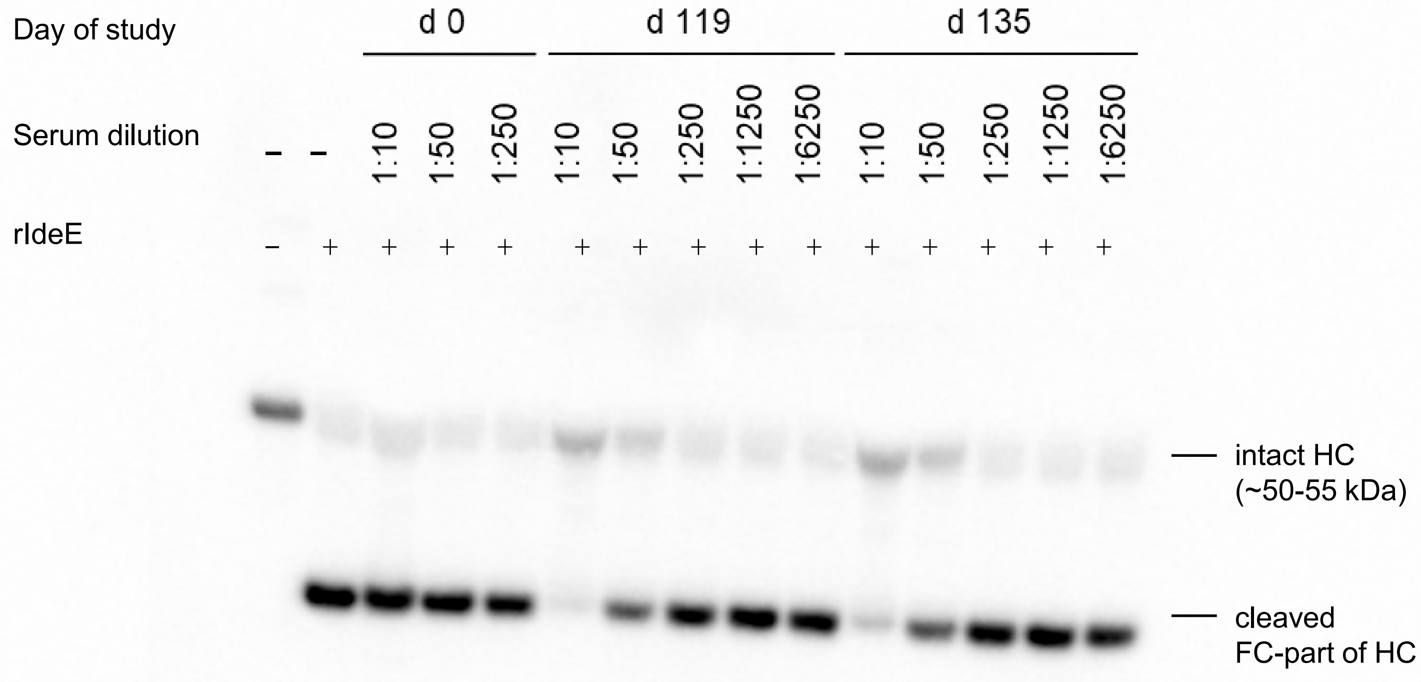

Pony 3161

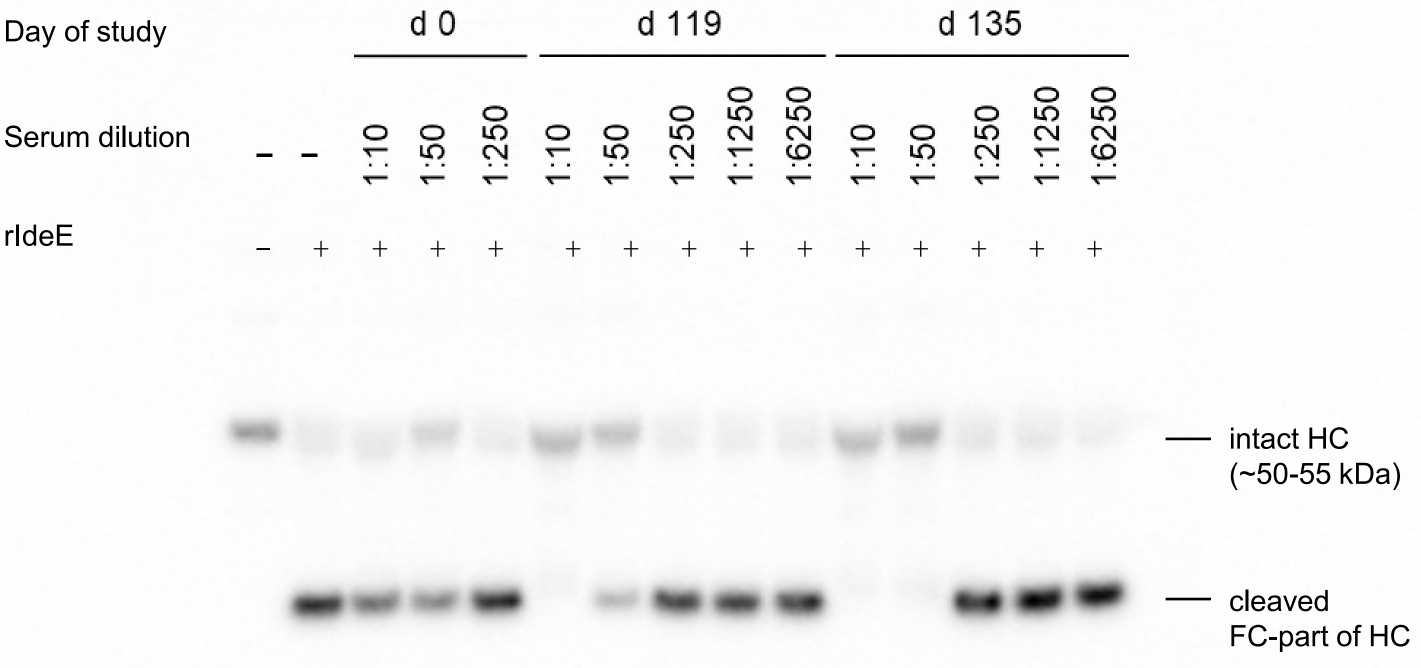

Exp I group 1

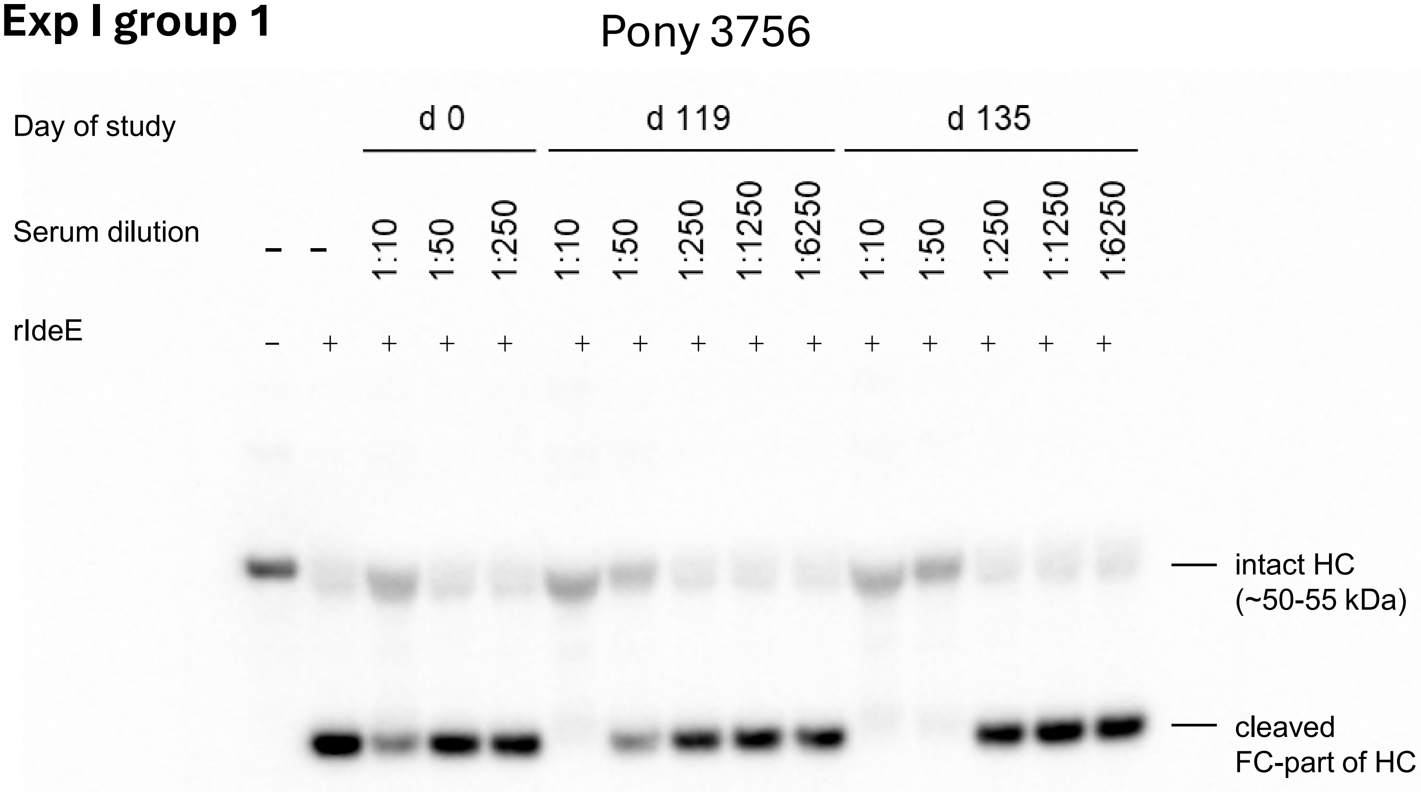
Pony 3756
Day of study
d 0
d 119
d 135
Serum dilution
–
–
1:10
1:50
1:250
1:10
1:50
1:250
1:1250
1:6250
1:10
1:50
1:250
1:1250
1:6250
rIdeE
–
+
+
+
+
+
+
+
+
+
+
+
+
+
+
intact HC
(~50-55 kDa)
cleaved
FC-part of HC

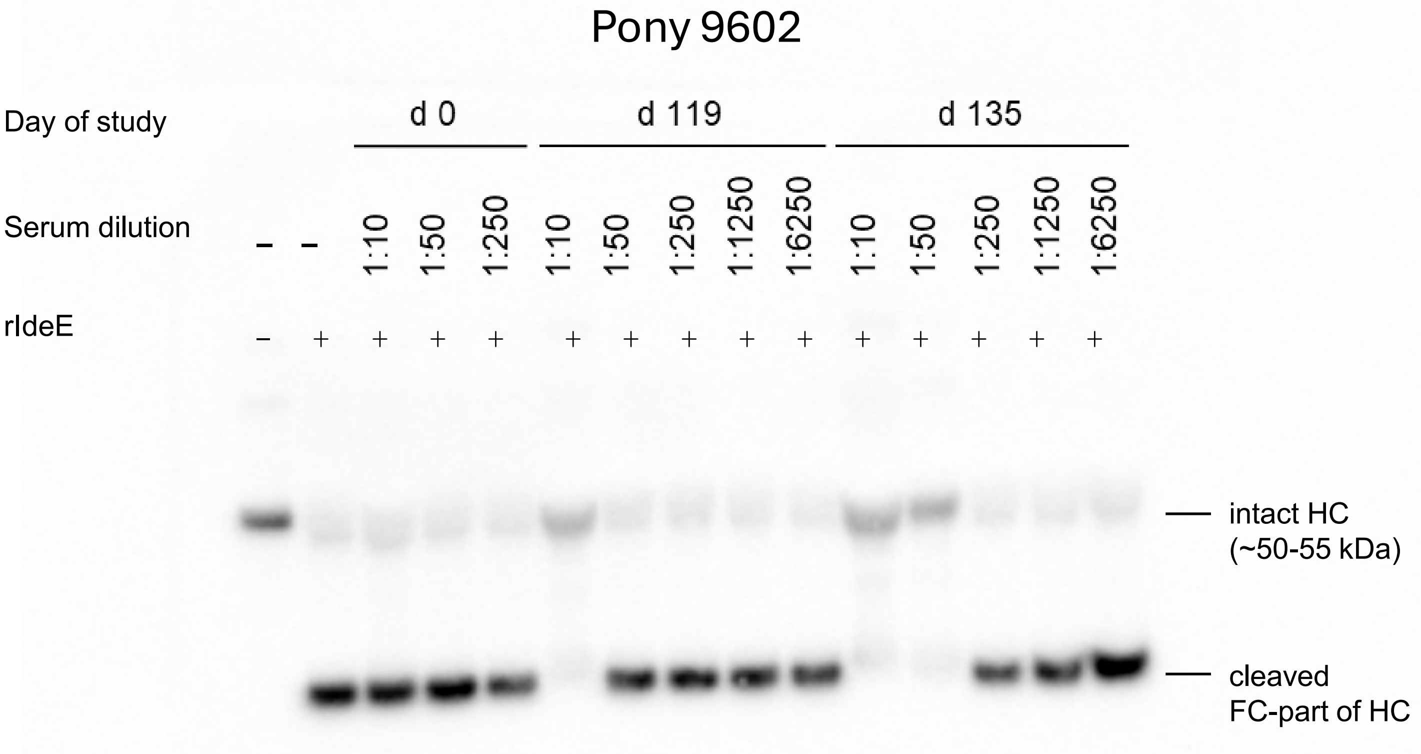
Pony 9602
Day of study
d 0
d 119
d 135
Serum dilution
–
–
1:10
1:50
1:250
1:10
1:50
1:250
1:1250
1:6250
1:10
1:50
1:250
1:1250
1:6250
rIdeE
–
+
+
+
+
+
+
+
+
+
+
+
+
+
+
intact HC
(~50-55 kDa)
cleaved
FC-part of HC

Exp I group 2

Pony 0079

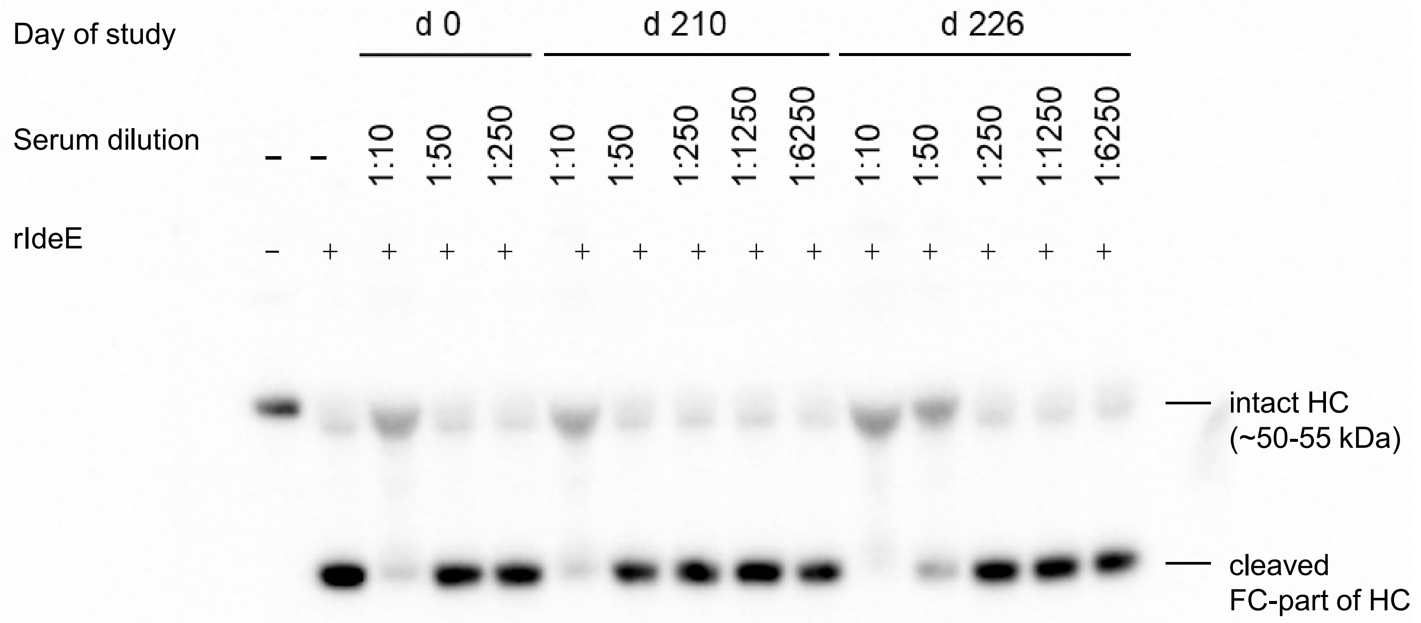

Pony 3038

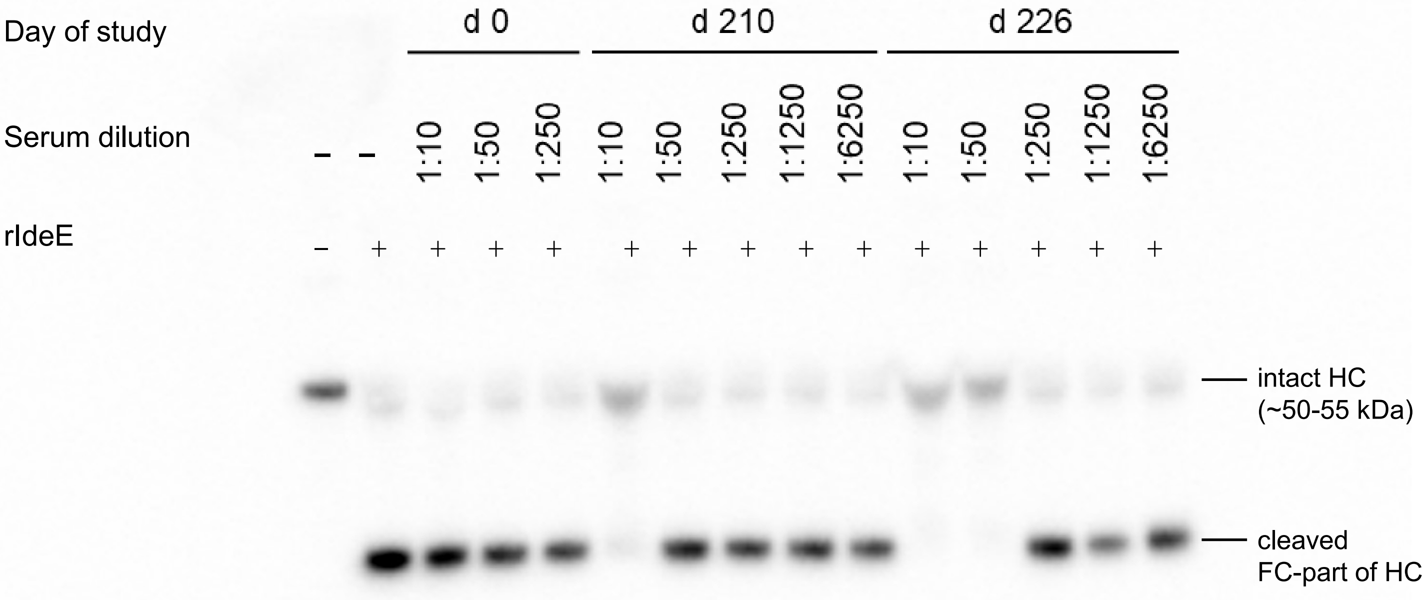

Exp I group 2

Pony 3150

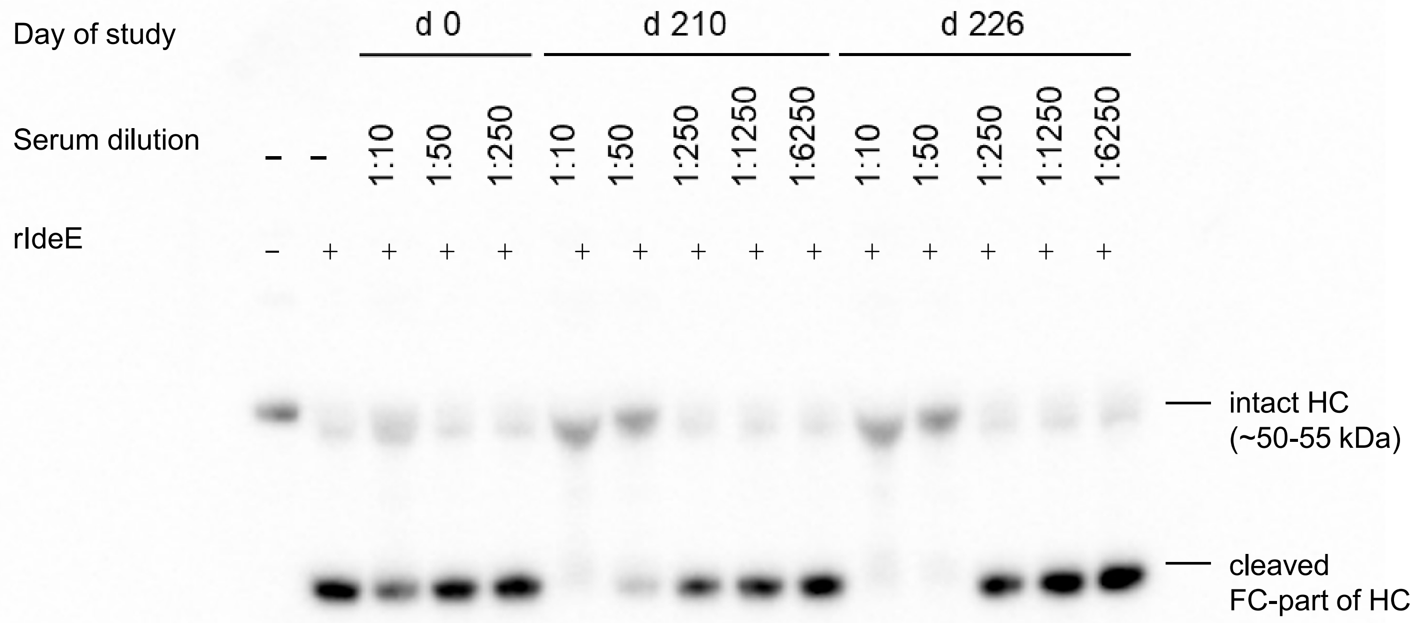

Pony 6593

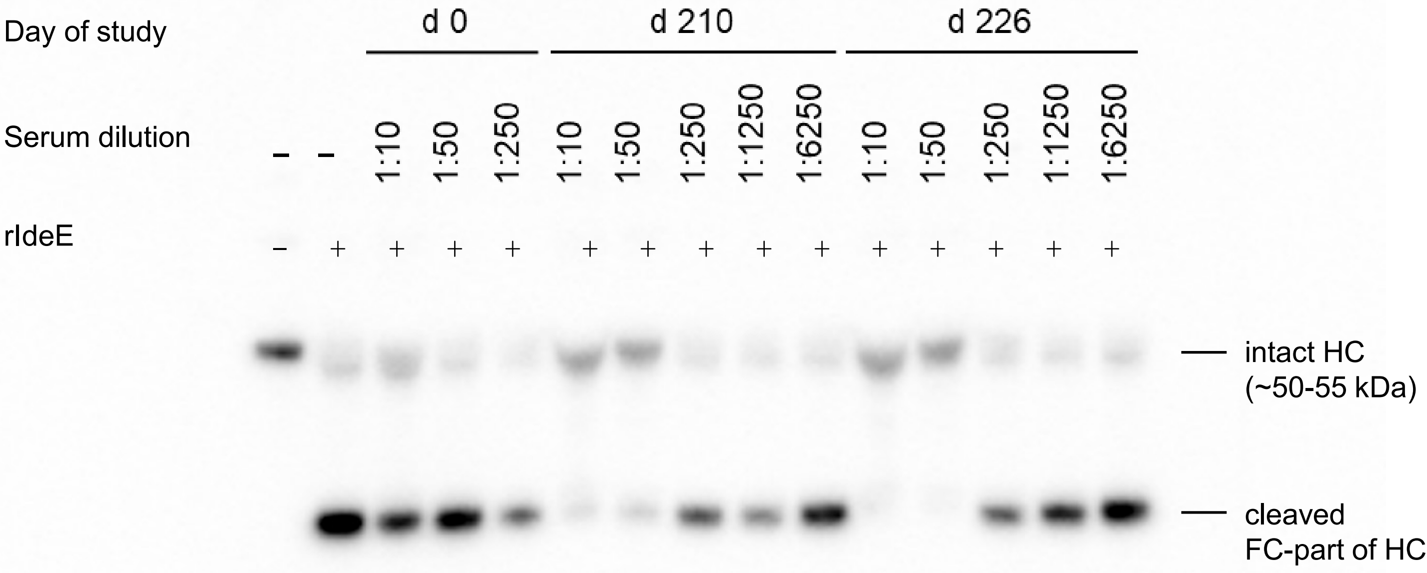

Exp I group 3

Pony 0016

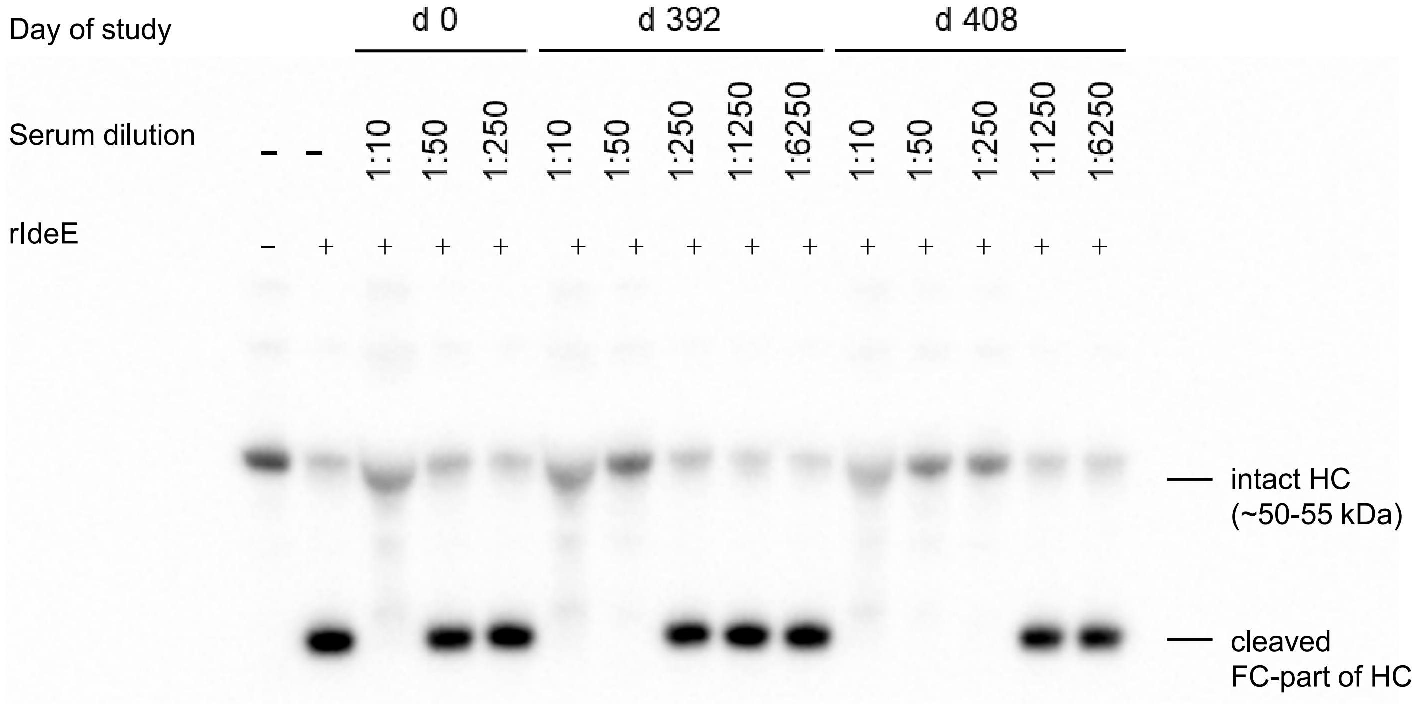

Pony 2015

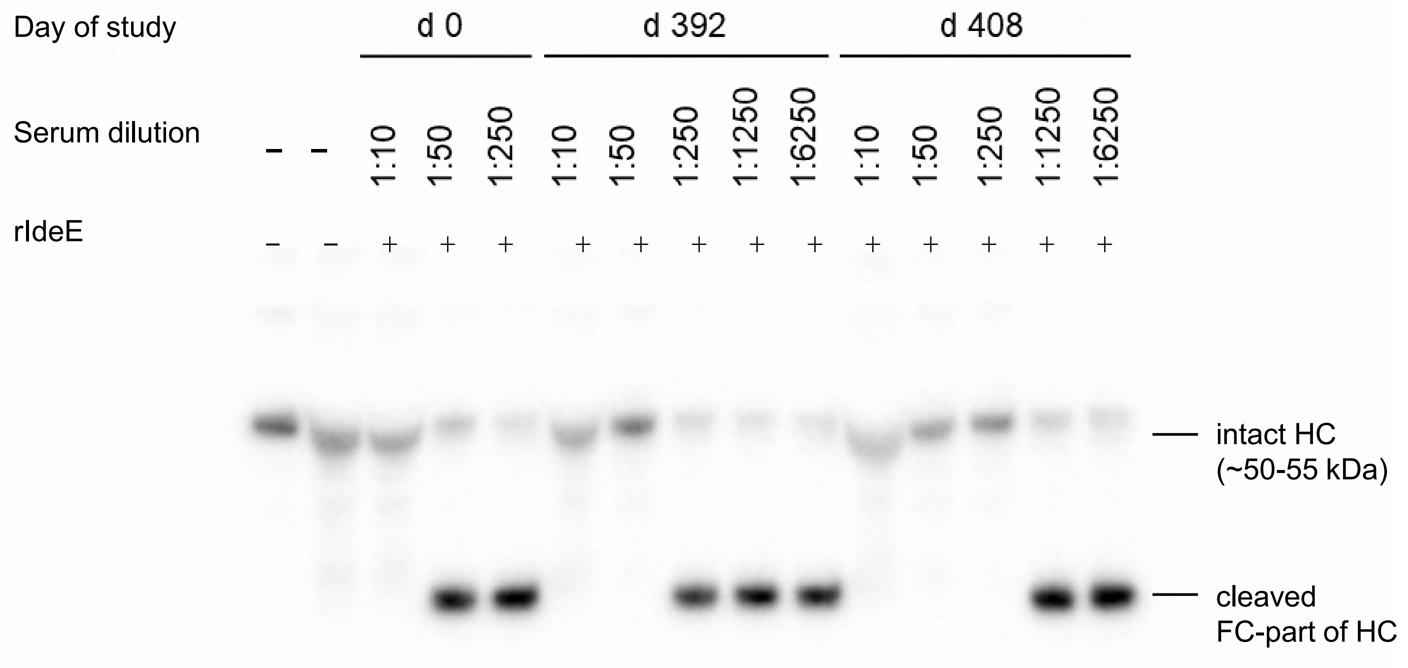

Exp I group 3

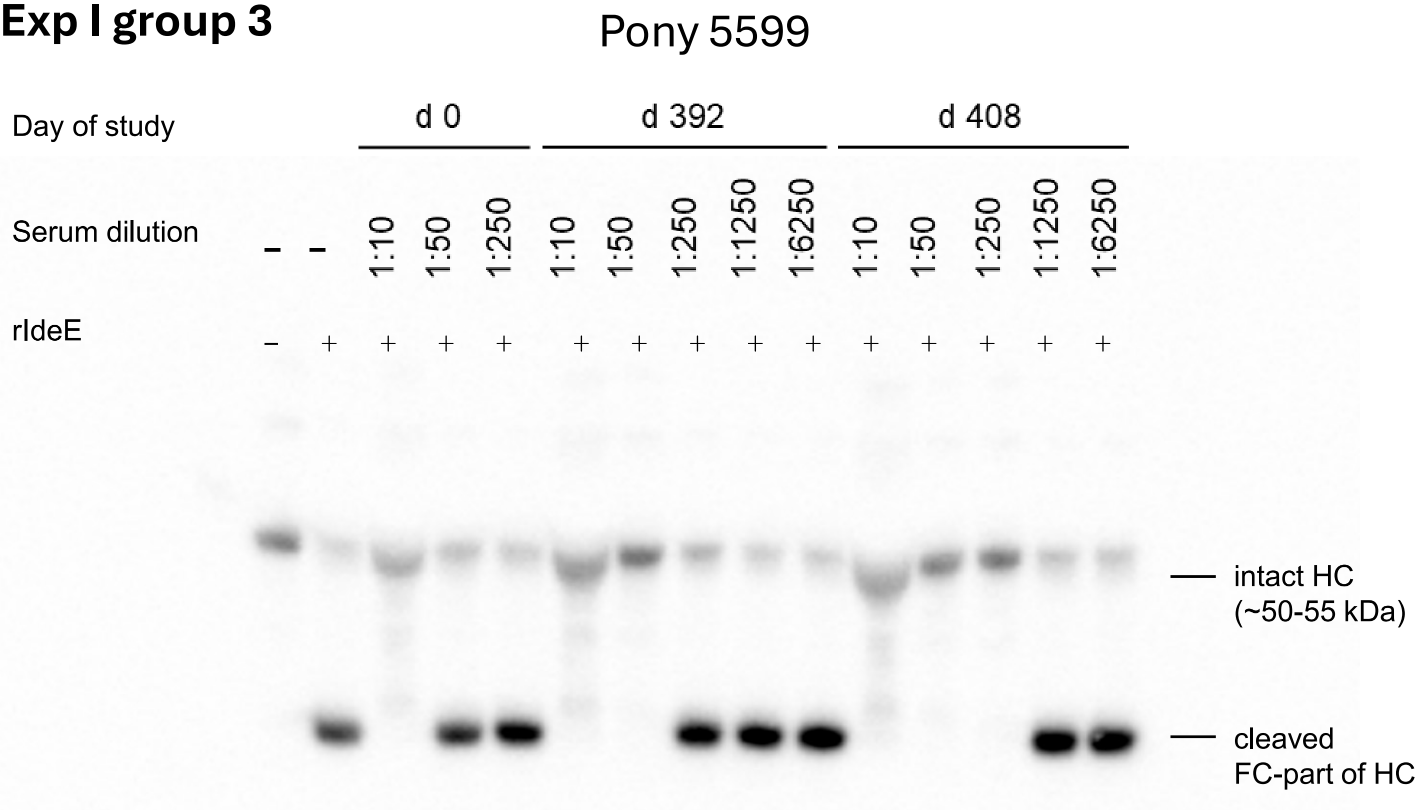
Pony 5599
Day of study
d 0
d 392
d 408
Serum dilution
1:10
1:50
1:250
1:10
1:50
1:250
1:1250
1:6250
1:10
1:50
1:250
1:1250
1:6250
rIdeE
intact HC (~50-55 kDa)
cleaved FC-part of HC

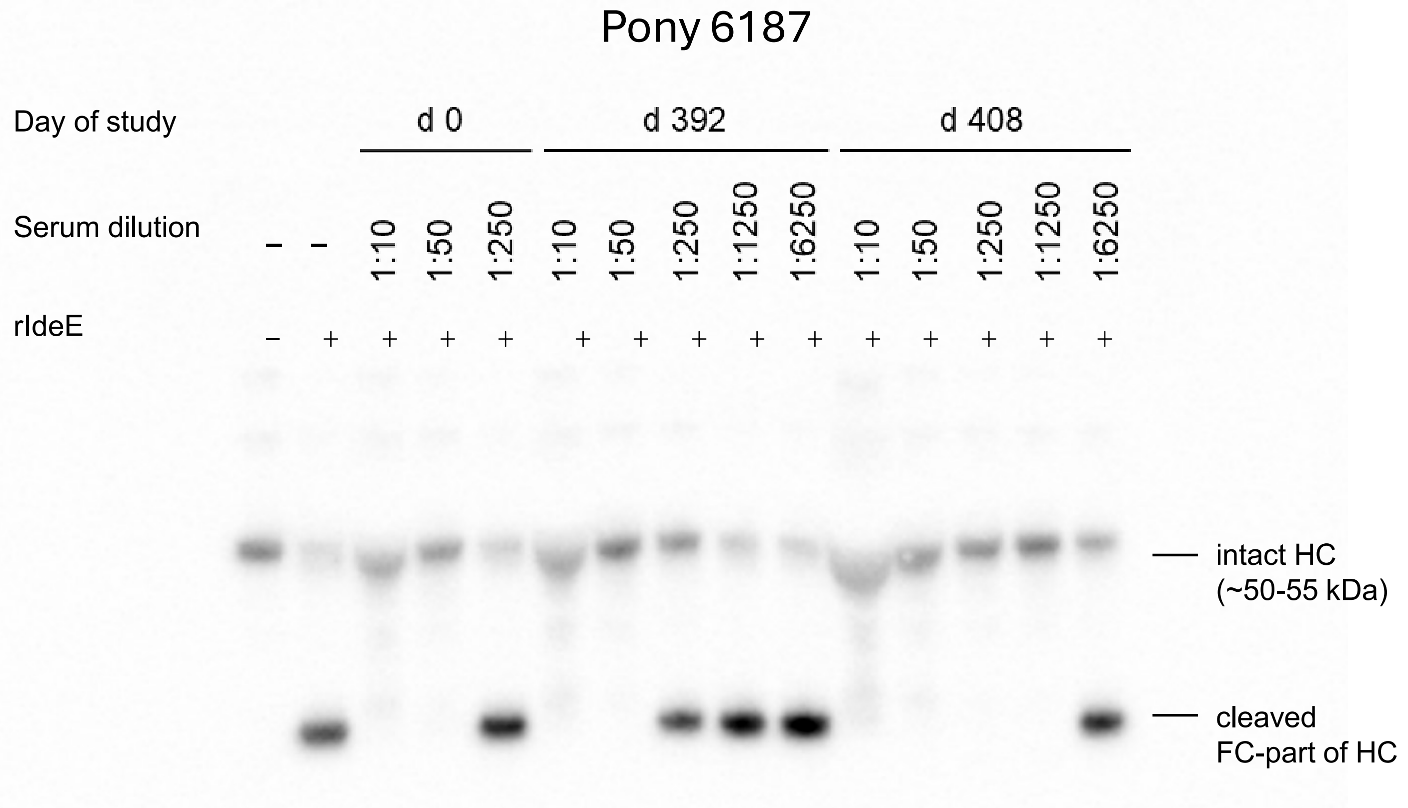
Pony 6187
Day of study
d 0
d 392
d 408
Serum dilution
1:10
1:50
1:250
1:10
1:50
1:250
1:1250
1:6250
1:10
1:50
1:250
1:1250
1:6250
rIdeE
intact HC (~50-55 kDa)
cleaved FC-part of HC

Exp I group 3 ext

Pony 0016

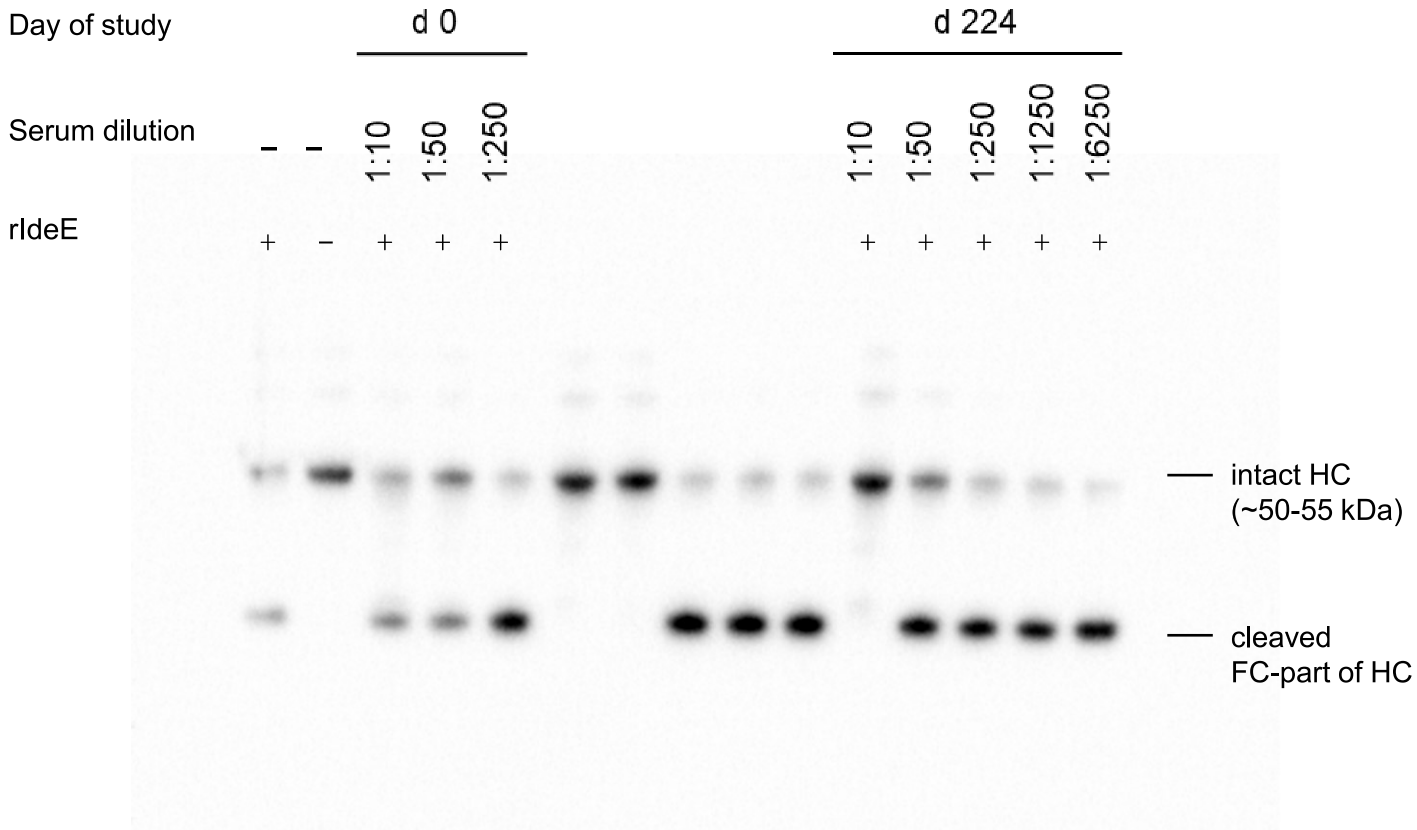

Pony 2015

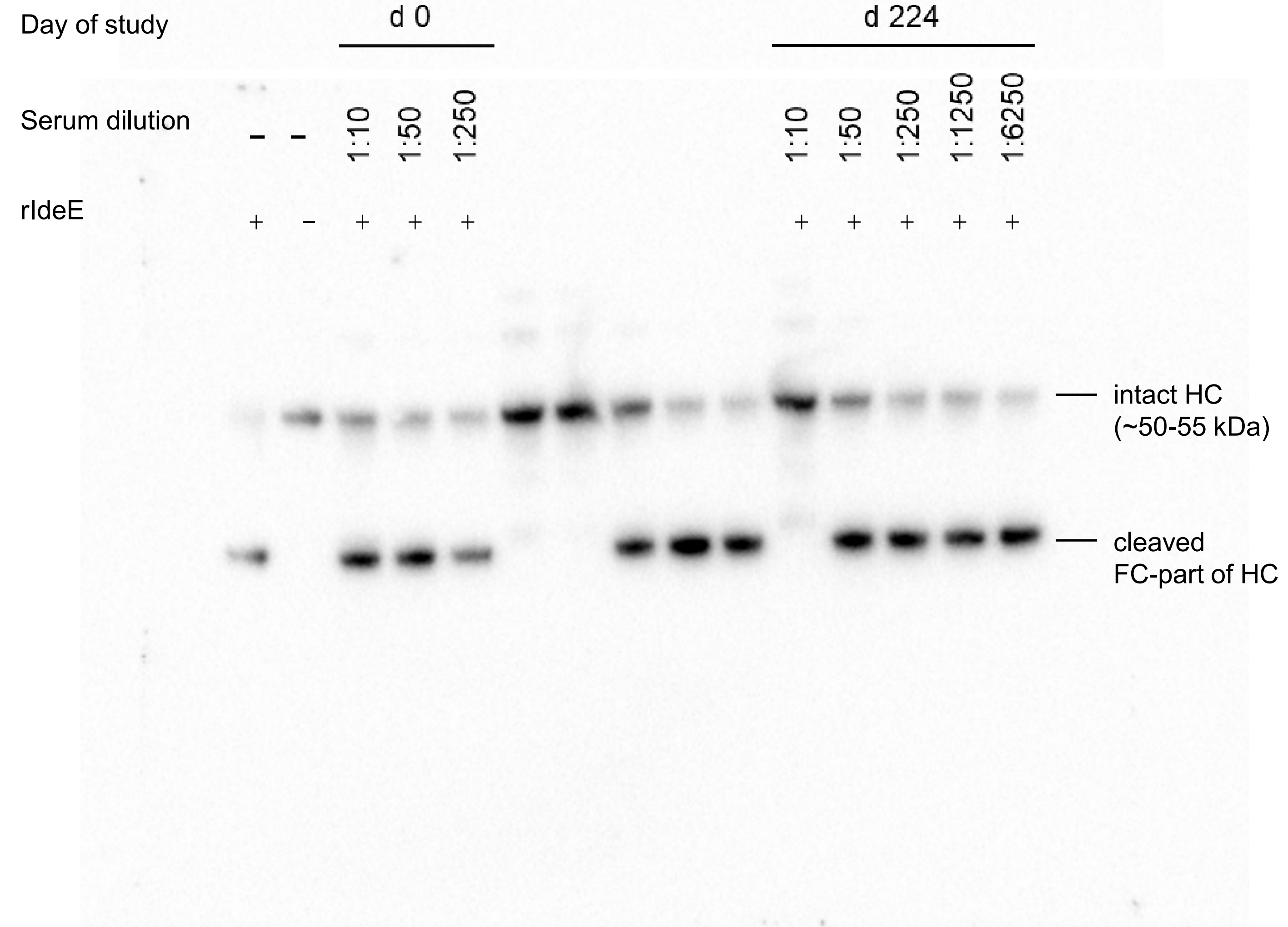

Exp I group 3 ext

Pony 5599

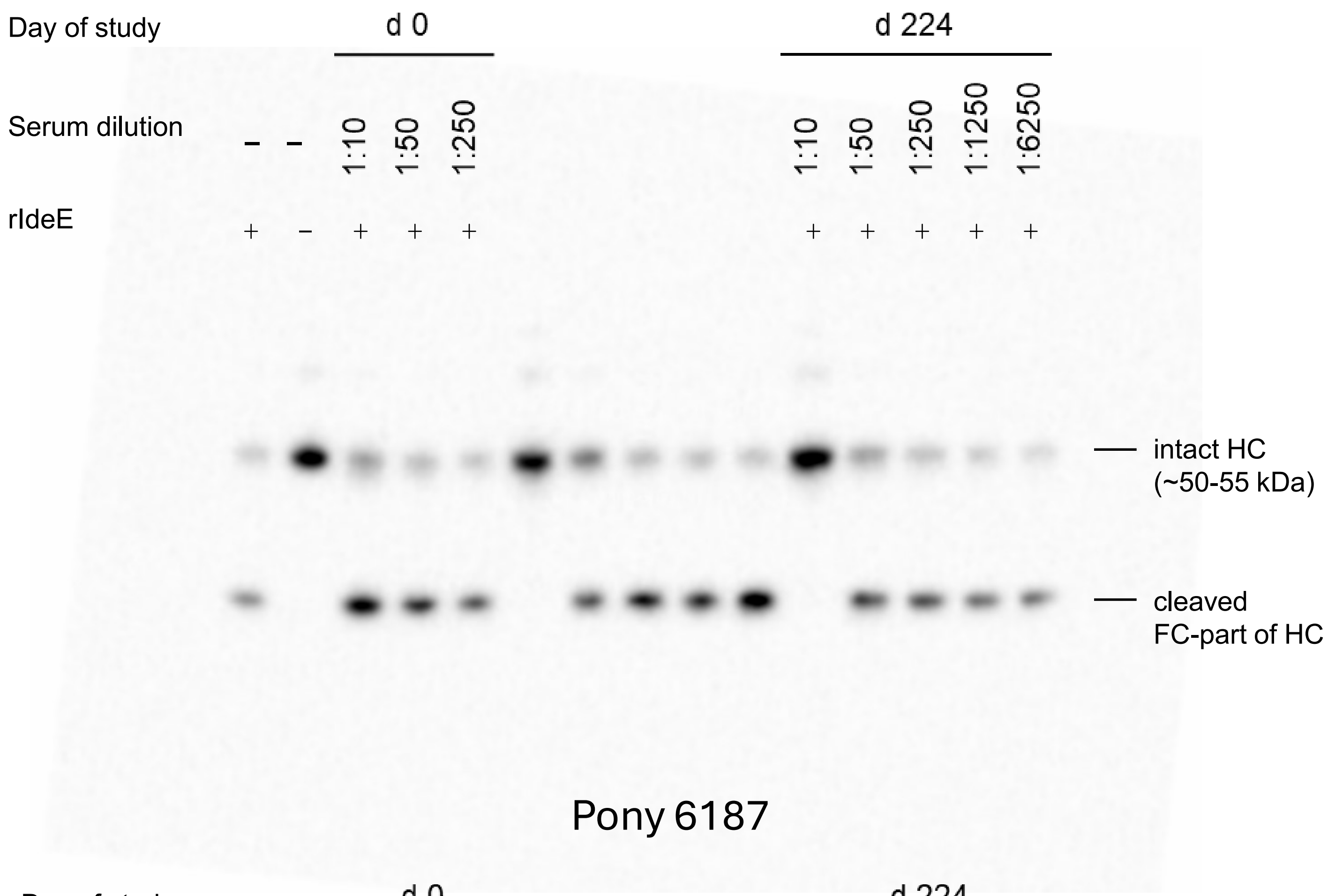

Pony 6187

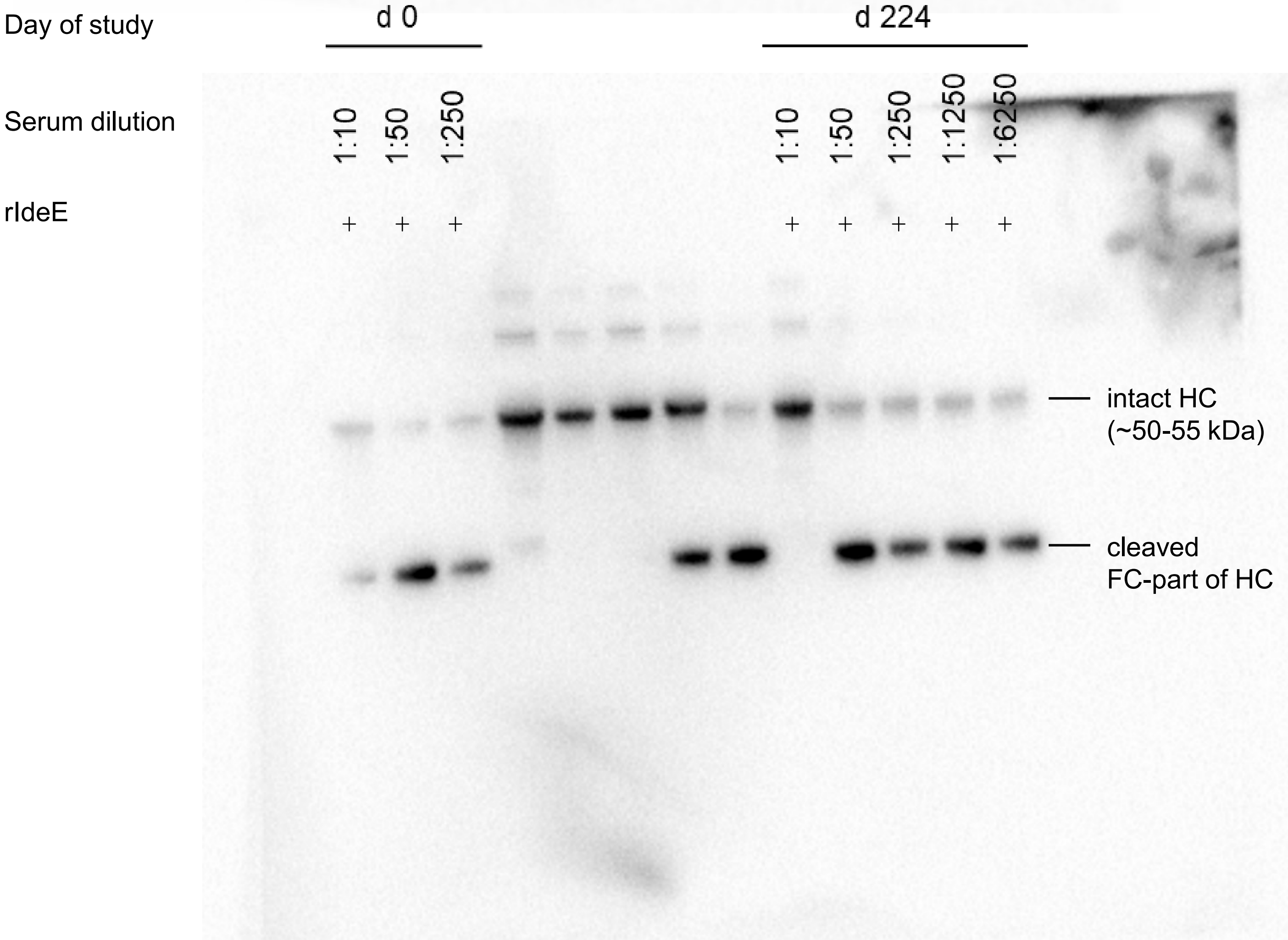

Exp IV

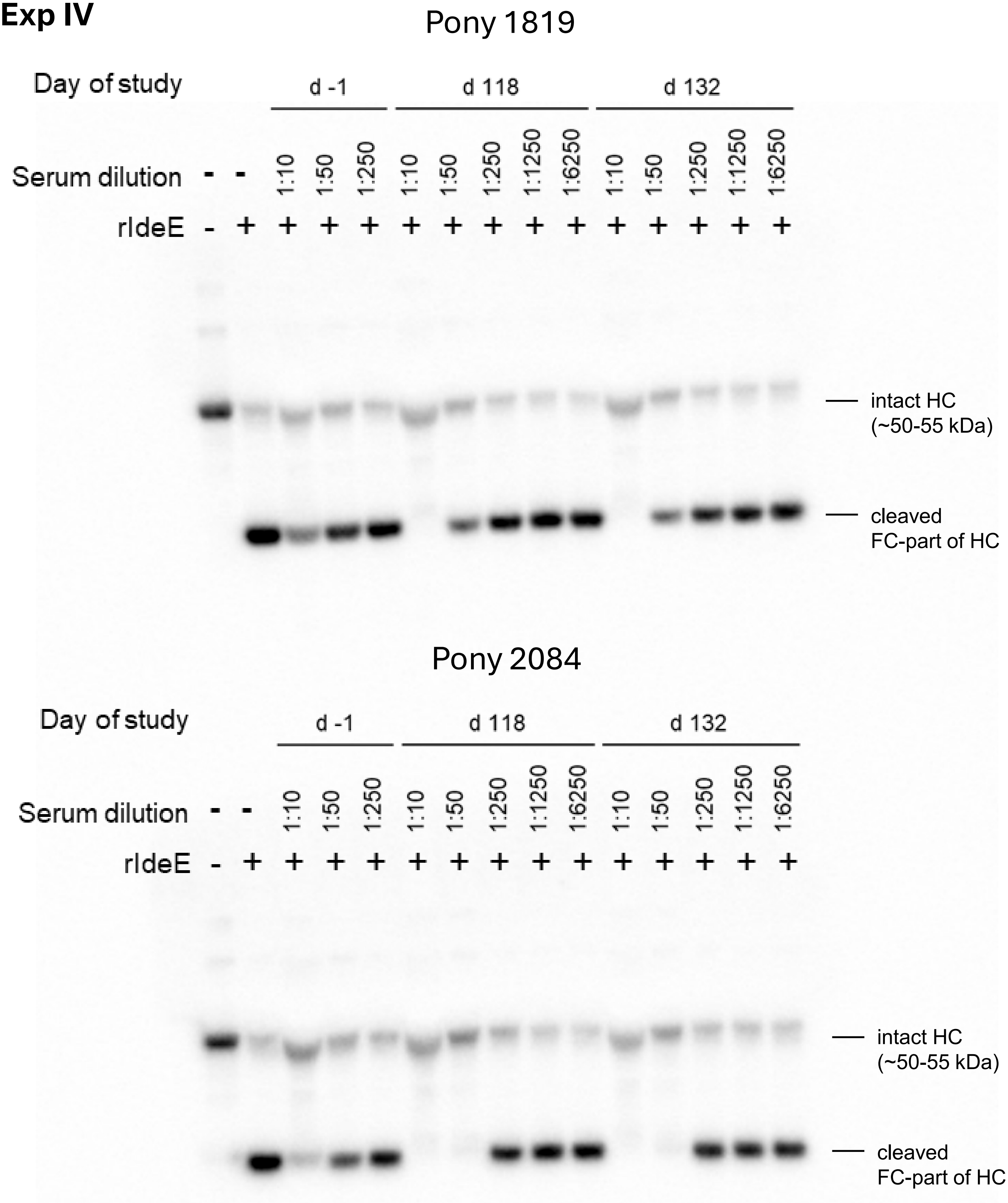
Pony 1819
Day of study
d -1
d 118
d 132
Serum dilution
- -
1:10 1:50 1:250 1:10 1:50 1:250 1:1250 1:6250 1:10 1:50 1:250 1:1250 1:6250
rIdeE
- + + + + + + + + + + + + + +
intact HC
(~50-55 kDa)
cleaved
FC-part of HC
Pony 2084
Day of study
d -1
d 118
d 132
Serum dilution
- -
1:10 1:50 1:250 1:10 1:50 1:250 1:1250 1:6250 1:10 1:50 1:250 1:1250 1:6250
rIdeE
- + + + + + + + + + + + + + +
intact HC
(~50-55 kDa)
cleaved
FC-part of HC

Exp IV

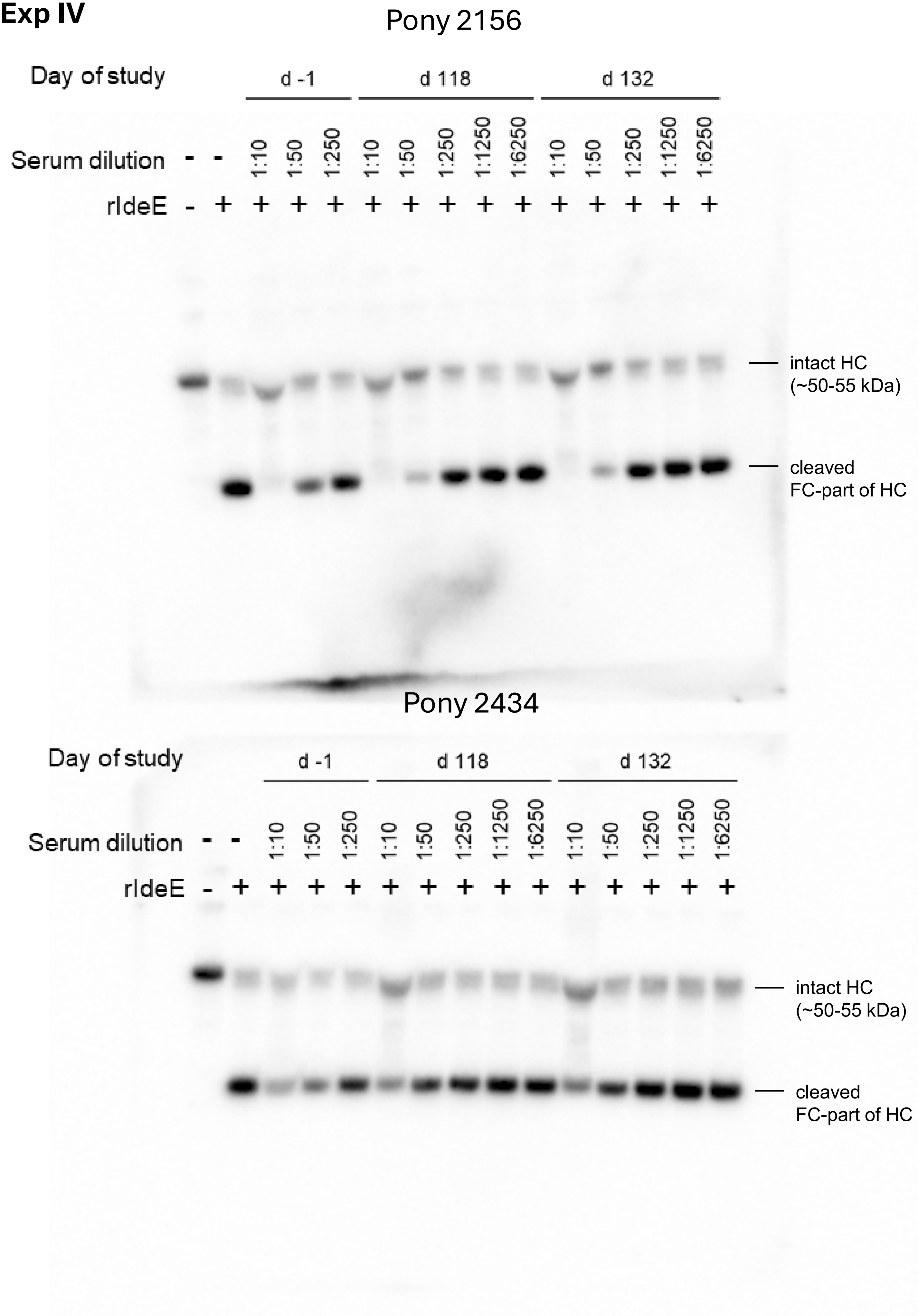

Pony 2156
Day of study
d -1
d 118
d 132
Serum dilution
- -
1:10
1:50
1:250
1:10
1:50
1:250
1:1250
1:6250
1:10
1:50
1:250
1:1250
1:6250
rIdeE
- + + + + + + + + + + + + + +
intact HC
(~50-55 kDa)
cleaved
FC-part of HC
Pony 2434
Day of study
d -1
d 118
d 132
Serum dilution
- -
1:10
1:50
1:250
1:10
1:50
1:250
1:1250
1:6250
1:10
1:50
1:250
1:1250
1:6250
rIdeE
- + + + + + + + + + + + + + +
intact HC
(~50-55 kDa)
cleaved
FC-part of HC

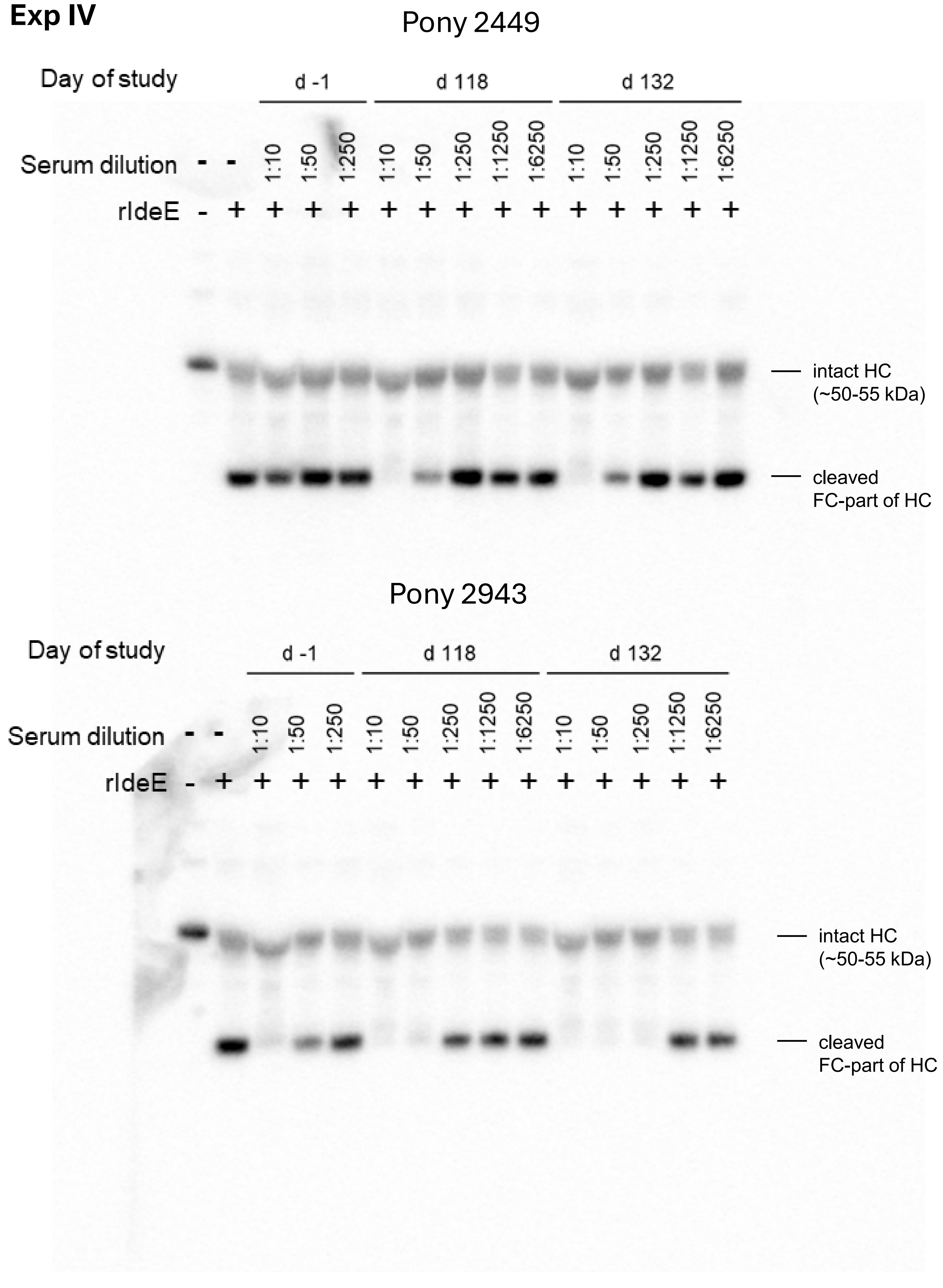
Exp IV
Pony 2449
Day of study
d -1
d 118
d 132
Serum dilution
-
-
1:10
1:50
1:250
1:10
1:50
1:250
1:1250
1:6250
1:10
1:50
1:250
1:1250
1:6250
rIdeE
-
+
+
+
+
+
+
+
+
+
+
+
+
+
+
intact HC (~50-55 kDa)
cleaved FC-part of HC
Pony 2943
Day of study
d -1
d 118
d 132
Serum dilution
-
-
1:10
1:50
1:250
1:10
1:50
1:250
1:1250
1:6250
1:10
1:50
1:250
1:1250
1:6250
rIdeE
-
+
+
+
+
+
+
+
+
+
+
+
+
+
+
intact HC (~50-55 kDa)
cleaved FC-part of HC

Exp IV

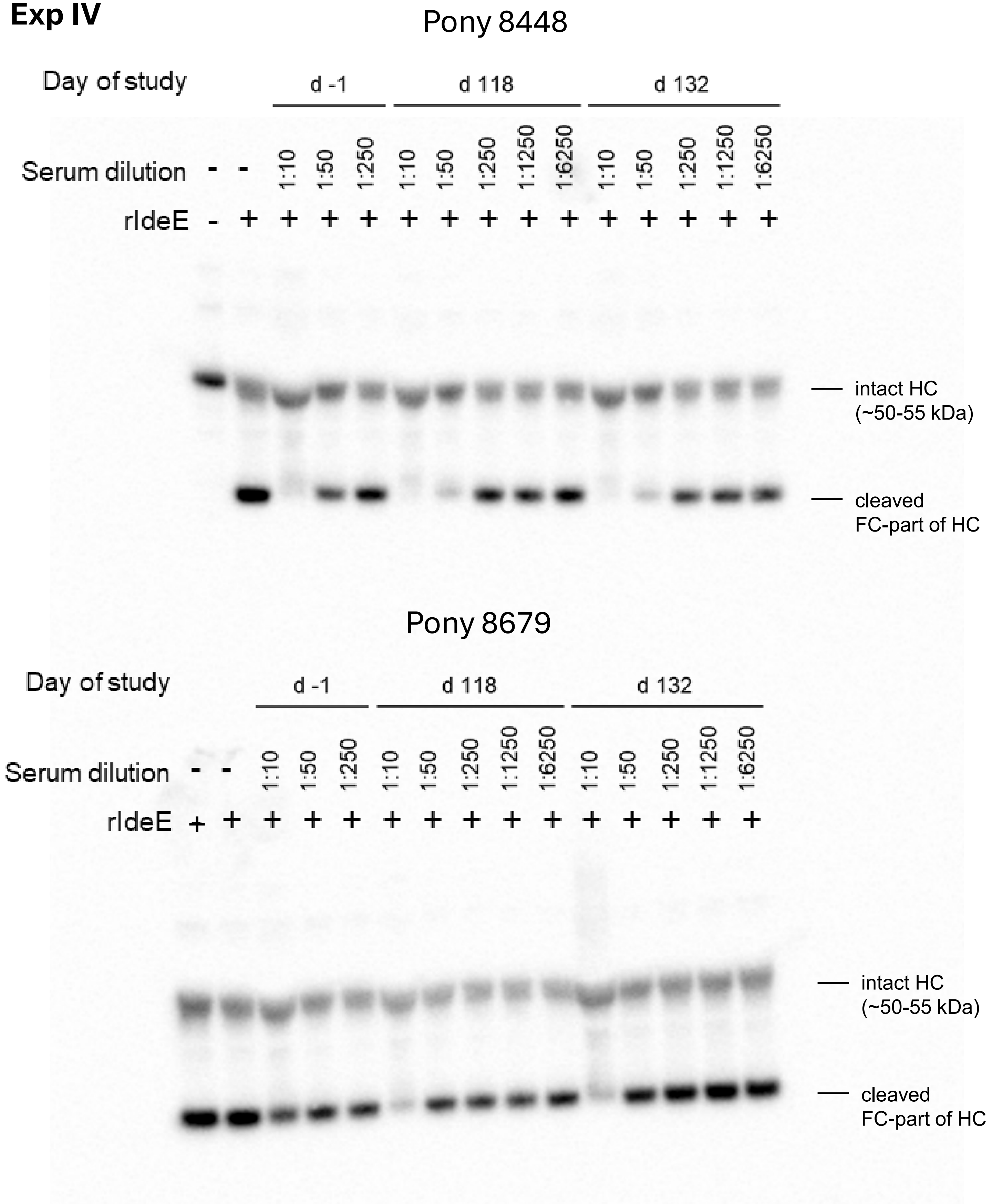
Pony 8448
Day of study
d -1
d 118
d 132
Serum dilution
- -
1:10 1:50 1:250 1:10 1:50 1:250 1:1250 1:6250 1:10 1:50 1:250 1:1250 1:6250
rIdeE
- + + + + + + + + + + + + + +
intact HC (~50-55 kDa)
cleaved FC-part of HC
Pony 8679
Day of study
d -1
d 118
d 132
Serum dilution
- -
1:10 1:50 1:250 1:10 1:50 1:250 1:1250 1:6250 1:10 1:50 1:250 1:1250 1:6250
rIdeE
+ + + + + + + + + + + + + + +
intact HC (~50-55 kDa)
cleaved FC-part of HC

Exp IV

Pony 1235

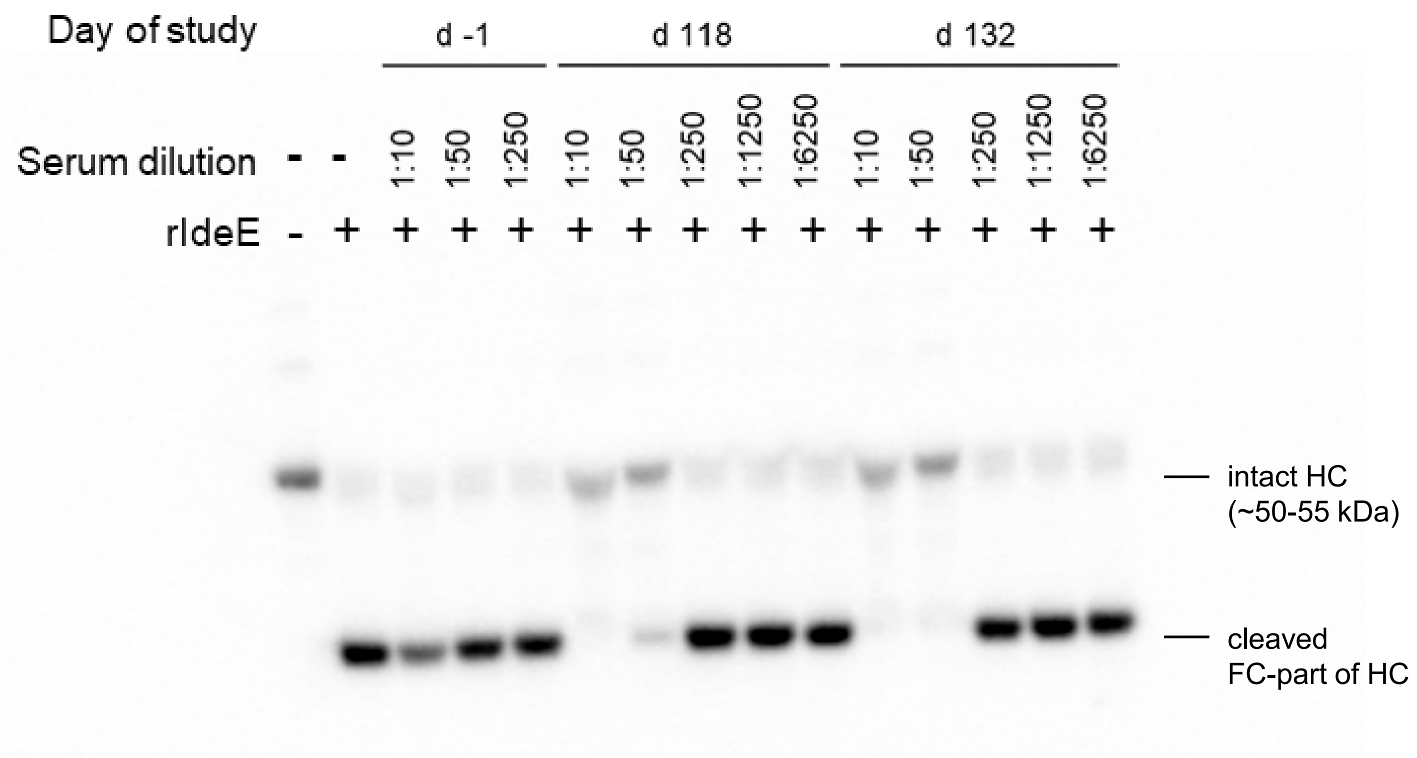

Pony 2726

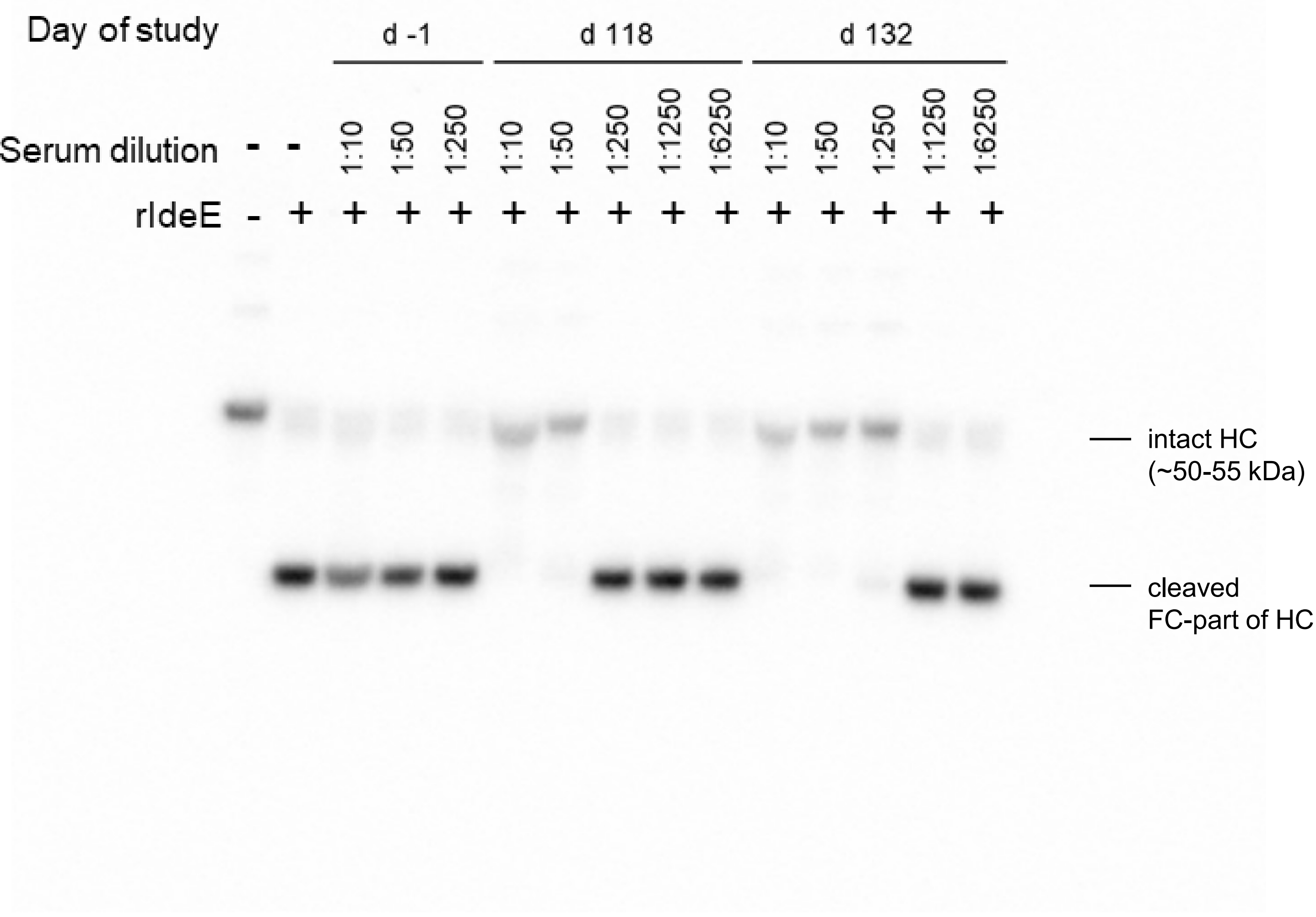

Exp IV

Pony 2997

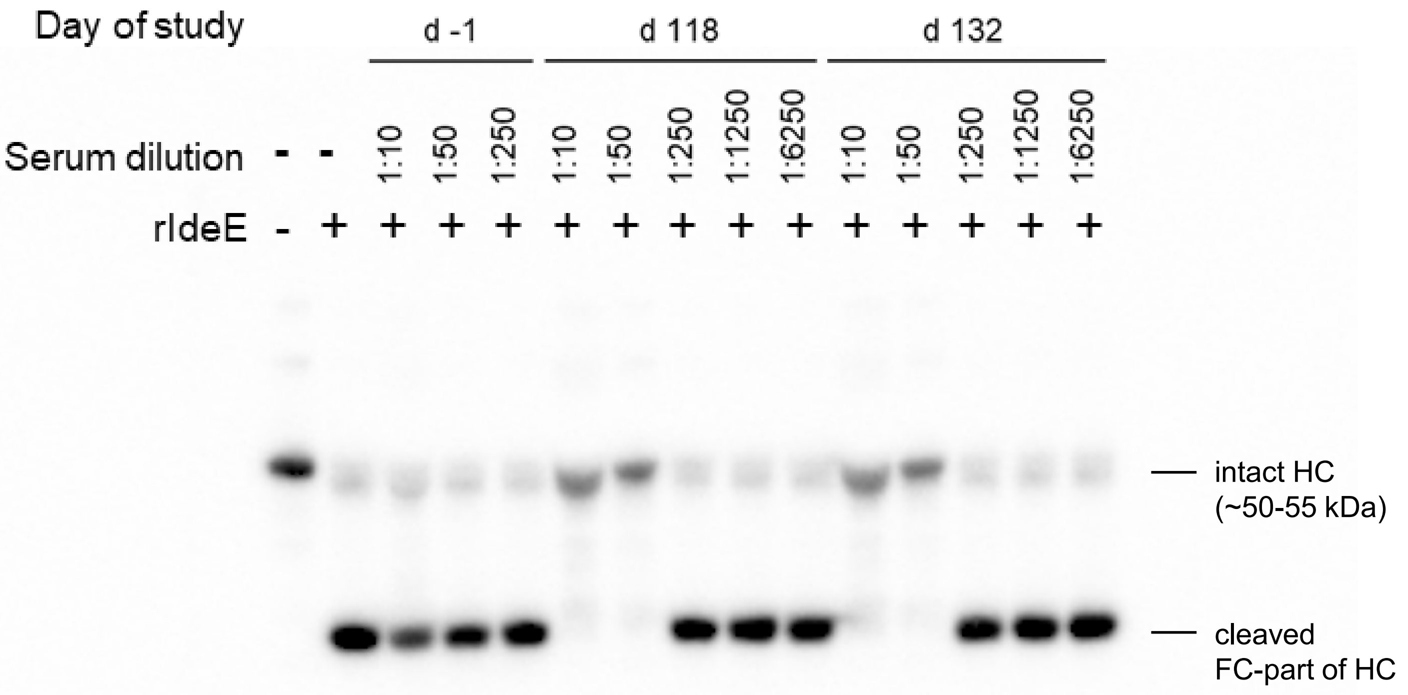

Pony 3121

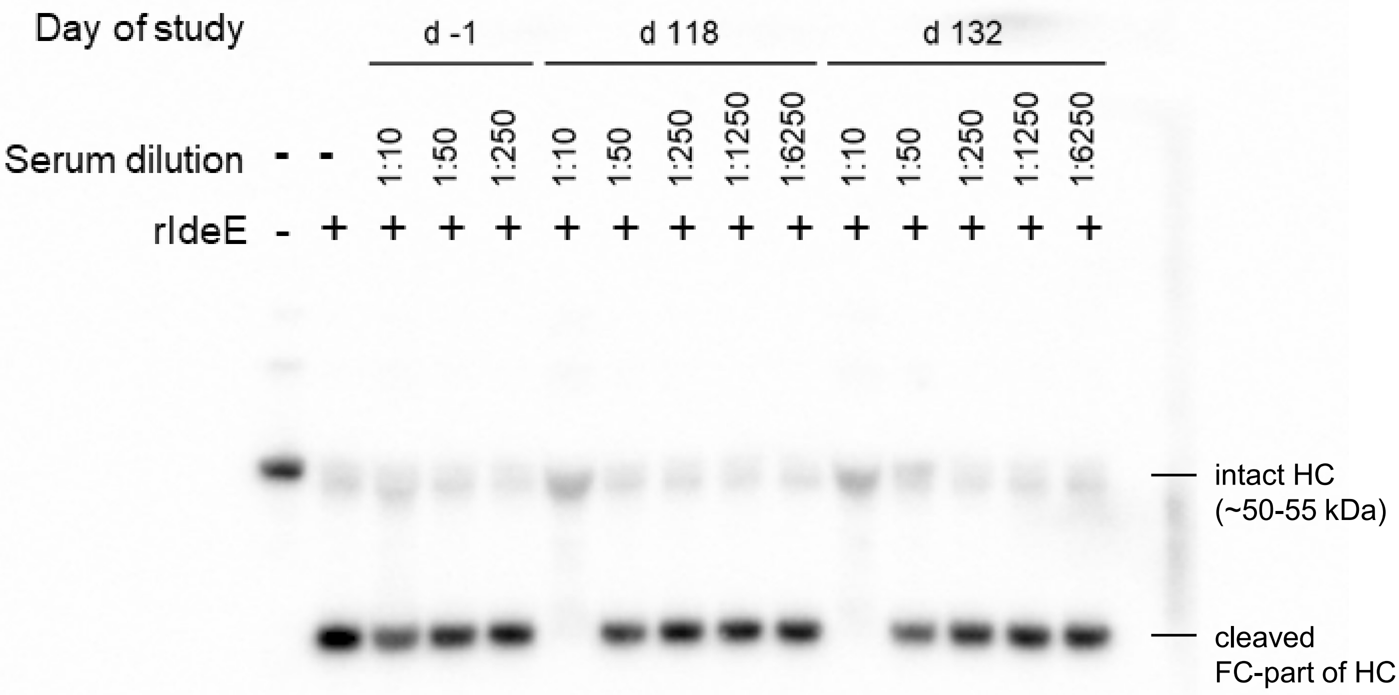

Exp IV

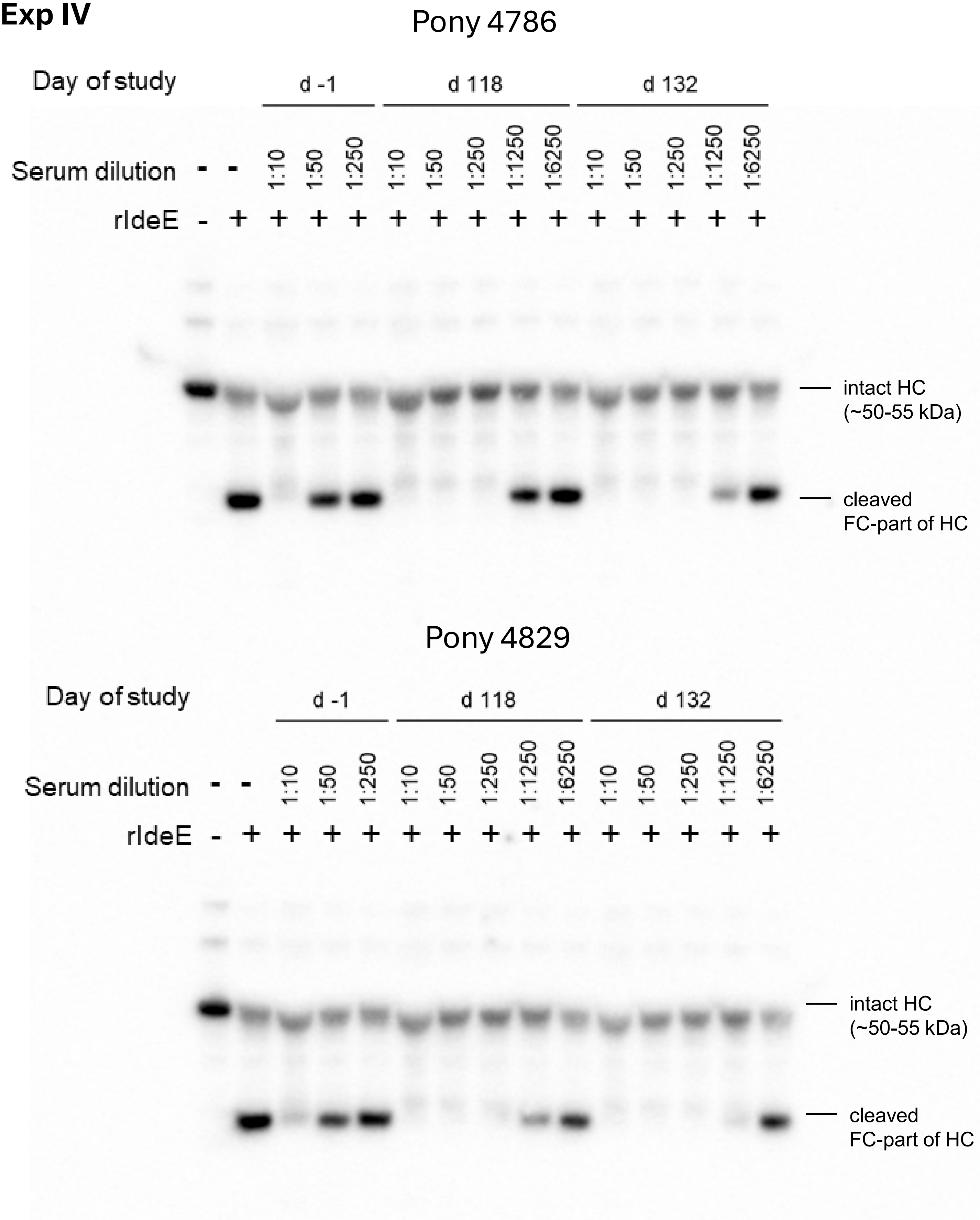
Pony 4786
Day of study
d -1
d 118
d 132
Serum dilution
- -
1:10 1:50 1:250 1:10 1:50 1:250 1:1250 1:6250 1:10 1:50 1:250 1:1250 1:6250
rIdeE
- + + + + + + + + + + + + + +
intact HC
(~50-55 kDa)
cleaved
FC-part of HC
Pony 4829
Day of study
d -1
d 118
d 132
Serum dilution
- -
1:10 1:50 1:250 1:10 1:50 1:250 1:1250 1:6250 1:10 1:50 1:250 1:1250 1:6250
rIdeE
- + + + + + + + + + + + + + +
intact HC
(~50-55 kDa)
cleaved
FC-part of HC

Exp IV

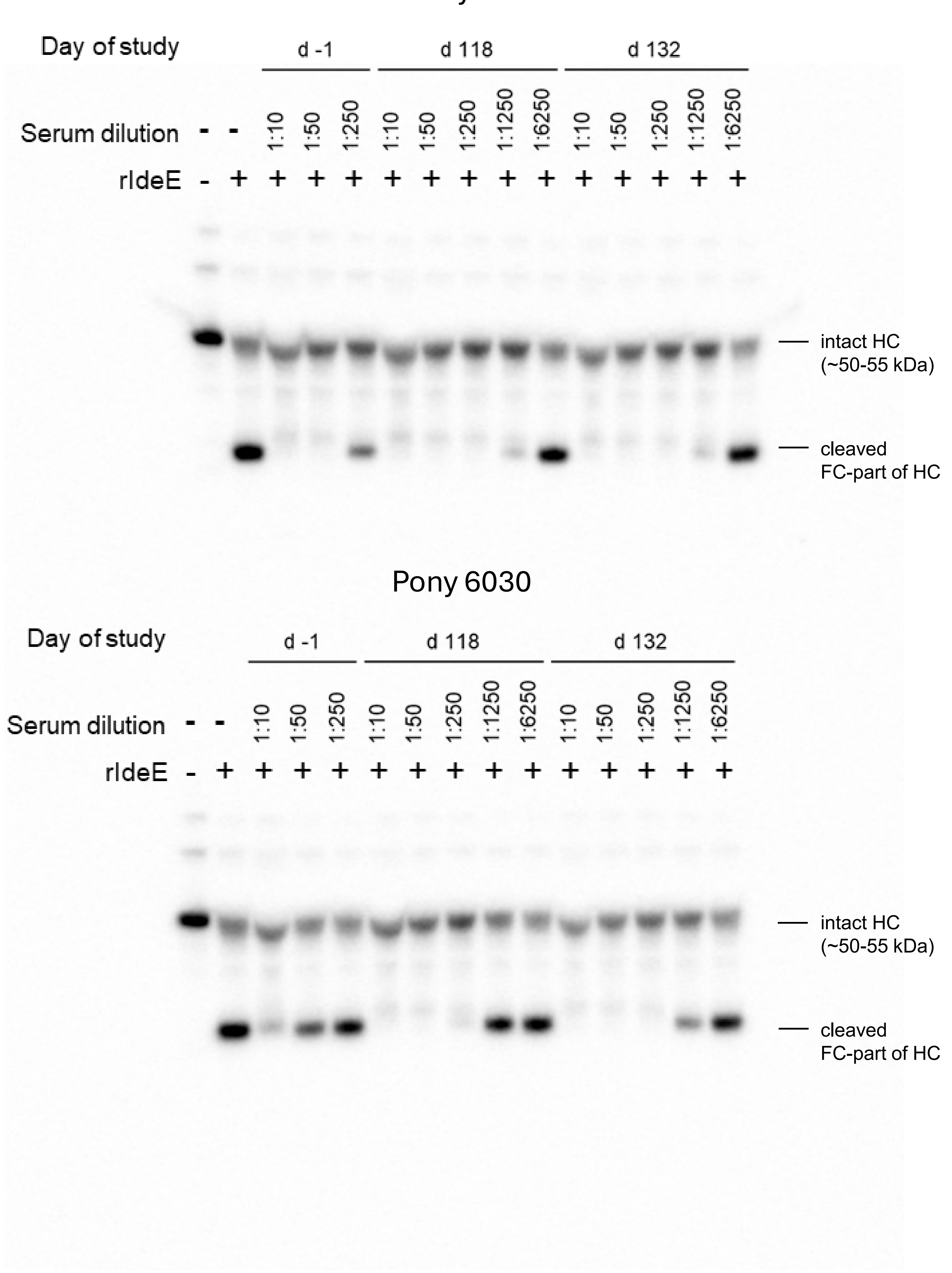
Pony 5305
Day of study
d -1
d 118
d 132
Serum dilution
- -
1:10 1:50 1:250 1:10 1:50 1:250 1:1250 1:6250 1:10 1:50 1:250 1:1250 1:6250
rIdeE
- + + + + + + + + + + + + + +
intact HC
(~50-55 kDa)
cleaved
FC-part of HC
Pony 6030
Day of study
d -1
d 118
d 132
Serum dilution
- -
1:10 1:50 1:250 1:10 1:50 1:250 1:1250 1:6250 1:10 1:50 1:250 1:1250 1:6250
rIdeE
- + + + + + + + + + + + + + +
intact HC
(~50-55 kDa)
cleaved
FC-part of HC
