# Supplementary material for: Neutralisation of the Immunoglobulin-Cleaving Activity of Streptococcus equi Subspecies equi IdeE by Blood Sera from Ponies Vaccinated with a Multicomponent Protein Vaccine
Source: Vaccines (Basel). 2025 Oct 17;13(10):1061. doi: 10.3390/vaccines13101061 (PMC12568127; doi:10.3390/vaccines13101061)
Supplement: Supplementary file 1 [file vaccines-13-01061-s001.zip › Suppl_File_S1_animal_welfare.pdf]

Animal welfare information

Further information:

Further details of the serology responses post-vaccination and the protection conferred against Experimental challenge in Experiment IV are provided in Robinson et al., 2020.

Ethical considerations:

Experiments I and IV were conducted under the auspices of a Home Office Project License according to the Animal Scientific Procedures Act 1986 and following ethical review and approval by the Animal Health Trust's Animal Welfare and Ethical Review Body (RPP 01_08, approved May 2008; reviewed and approved December 2012).

Animal housing and management:

All ponies were housed on the Animal Health Trust premises prior to, and during the Experiments according to AHT/SOP/EQU/04 - Routine Husbandry Procedures for Ponies.

Ponies in Experiments I and IV were kept at pasture on grass or in suitable barns with nearby facilities for sampling and vaccination. Drinking water was available *ad libitum* and nutrition was provided in an amount necessary to keep the ponies in good condition. Regular veterinary health checks with certification were conducted.

Experiment IV was a randomised, double-blinded and placebo controlled study on the efficacy of vaccination against *S. equi* challenge. Following the vaccination period, ponies were moved to the Allen Centre for Veterinary Studies (a purpose-built containment facility at the Animal Health Trust) prior to challenge. Ponies were separated randomly into four

different rooms. A maximum of eight ponies per room (6.4 m^2 per pony) was adhered to. Drinking water was provided *ad libitum* and nutrition was provided as necessary to keep the ponies in good condition. Each animal room was enriched by the use of long fibre feed (Haylage), mineral licks, molasses and flavoured licks, play balls, feed balls, radio and maintaining contact with members of their original peer group. The temperature of each room was maintained in accordance with Home Office Recommendations.

Health and welfare monitoring protocols:

During the vaccination phase of Experiments I and IV, clinical observations for the occurrence of local and/or systemic reactions were performed prior to and after vaccination according to SOP/EQU/08 (Pony Clinical Observations).

All ponies were examined daily for three days pre-vaccination, for 14 days post each vaccination and, in Experiment IV, for three days pre-challenge. Animals showing signs of ill health were examined at the earliest opportunity to establish a diagnosis.

Following challenge and for up to 21 days, each pony was examined once daily in the morning for the occurrence of clinical signs associated with *S. equi* infection and scored according to Robinson et al., 2020. A second visual examination was also performed in the afternoon for the purposes of animal welfare.

Animal welfare endpoints:

In Experiment IV, the humane end-point of pyrexia and a preference for haylage and water over dried pelleted food was used post-challenge. Ponies were euthanased on reaching the humane end-point and prior to the onset of more severe clinical signs or complications associated with *S. equi* infection.

Fate of experimental animals:

All 12 ponies in Experiment I completed the study and were released to the Animal Health Trust's pony herd.

All of the ponies in Experiment IV completed the vaccination phase of this study and went on to be challenged with *S. equi* strain 4047 as described previously (Robinson et al., 2020). Placebo ponies 2084, 2156, 2434, 2449, 2943 and 8679 and vaccinated pony 1235 were euthanased on reaching the humane end-point on day 11, 8, 9, 10, 8, 14 and 9 post-challenge, respectively. Placebo ponies 1819 and 8448 and vaccinated ponies 2726, 2997, 3121, 4786, 4829, 5305 and 6030 were euthanased on reaching the end of the study at 21 days post-challenge (Robinson et al., 2020).

Reference:

Robinson, C., Waller, A.S., Frykberg, L., Flock, M., Zachrisson, O., Guss, B., Flock, J.I., 2020. Intramuscular vaccination with Strangvac is safe and induces protection against equine strangles caused by *Streptococcus equi*. Vaccine 38, 4861-4868.
