# Supplementary material for: Neutralisation of the Immunoglobulin-Cleaving Activity of Streptococcus equi Subspecies equi IdeE by Blood Sera from Ponies Vaccinated with a Multicomponent Protein Vaccine
Source: Vaccines (Basel). 2025 Oct 17;13(10):1061. doi: 10.3390/vaccines13101061 (PMC12568127; doi:10.3390/vaccines13101061)
Supplement: Supplementary file 1 [file vaccines-13-01061-s001.zip › Suppl_tab_S1_titres.pdf]

Supplementary table 1: Summary table of the IdeE neutralisation titres and IdeE-specific antibody titres.

Experiment 1 group 1	IdeE neutralisation titre			Log_{10} IdeE neutralisation titre			Log_{10} IdeE-specific antibody titre*		
	Vaccination Period			Vaccination Period			Vaccination Period		
	Pre-V1	Pre-V3	Post-V3	Pre-V1	Pre-V3	Post-V3	Pre-V1	Pre-V3	Post-V3
**Day**	0	119	135	0	119	135	0	119	135
**Pony number**									
292	2	10	10	0.30	1.00	1.00	2.20	3.94	4.67
3161	2	50	50	0.30	1.70	1.70	2.82	4.19	4.92
3756	2	10	50	0.30	1.00	1.70	3.41	4.19	4.88
9602	2	10	50	0.30	1.00	1.70	2.70	4.00	4.81

Experiment 1 group 2	IdeE neutralisation titre			Log_{10} IdeE neutralisation titre			Log_{10} IdeE-specific antibody titre*		
	Vaccination Period			Vaccination Period			Vaccination Period		
	Pre-V1	Pre-V3	Post-V3	Pre-V1	Pre-V3	Post-V3	Pre-V1	Pre-V3	Post-V3
**Day**	0	210	226	0	210	226	0	210	226
**Pony number**									
79	10	10	50	1.00	1.00	1.70	3.91	3.98	5.00
3038	2	10	50	0.30	1.00	1.70	2.69	3.89	4.93
3150	2	50	50	0.30	1.70	1.70	3.48	4.15	5.02
6593	2	50	50	0.30	1.70	1.70	2.99	3.95	5.14

Experiment 1 group 3						
**Day**	0	224	0	224	0	224
**Pony number**						
0016	2	10	0.30	1.00	2.85	3.85
2015	2	10	0.30	1.00	3.05	3.65
5599	2	10	0.30	1.00	2.90	3.65
6187	10	10	1.00	1.00	3.35	4.05

Experiment 1 group 3	IdeE neutralisation titre			Log_{10} IdeE neutralisation titre			Log_{10} IdeE-specific antibody titre*		
	Vaccination Period			Vaccination Period			Vaccination Period		
	Pre-V1	Pre-V3	Post-V3	Pre-V1	Pre-V3	Post-V3	Pre-V1	Pre-V3	Post-V3
**Day**	0	392	408	0	392	408	0	392	408
**Pony number**									
0016	10	50	250	1.00	1.70	2.40	2.85	4.05	4.95
2015	10	50	250	1.00	1.70	2.40	3.05	3.55	4.20
5599	10	50	250	1.00	1.70	2.40	2.90	3.75	4.55
6187	50	50	1250	1.70	1.70	3.10	3.35	3.95	4.70

Experiment IV	IdeE neutralisation titre			Log_{10} IdeE neutralisation titre			Log_{10} IdeE-specific antibody titre*		
	Vaccination Period			Vaccination Period			Vaccination Period		
	Pre-V1	Pre-V3	Post-V3	Pre-V1	Pre-V3	Post-V3	Pre-V1	Pre-V3	Post-V3
**Day**	-1	118	132	-1	118	132	-1	118	132
**Control pony number**									
1819	2	10	10	0.30	1.00	1.00	2.54	3.00	2.93
2084	10	50	50	1.00	1.70	1.70	2.97	3.51	3.44
2156	10	50	50	1.00	1.70	1.70	2.93	3.39	3.25
2434	2	10	10	0.30	1.00	1.00	2.42	2.64	2.66
2449	2	50	50	0.30	1.70	1.70	2.47	2.76	2.67
2943	50	50	250	1.70	1.70	2.40	2.60	3.07	3.12
8448	50	50	50	1.70	1.70	1.70	3.08	2.90	2.80
8679	2	10	10	0.30	1.00	1.00	2.46	2.21	2.14
**Vaccinated pony number**									
1235	2	50	50	0.30	1.70	1.70	2.76	3.99	4.78
2726	2	50	250	0.30	1.70	2.40	2.53	3.98	4.92
2997	2	50	50	0.30	1.70	1.70	2.82	4.32	4.87
3121	2	10	10	0.30	1.00	1.00	2.53	3.88	4.53
4786	10	250	1250	1.00	2.40	3.10	3.21	4.66	5.07
4829	10	1250	1250	1.00	3.10	3.10	3.18	4.52	4.79
5303	250	1250	1250	2.40	3.10	3.10	3.57	4.44	4.83
6030	10	250	1250	1.00	2.40	3.10	2.80	4.29	4.81

*Log_{10} IdeE-specific antibody titre are imported from Robinson *et al.* , 2020.
