# Supplementary material for: Neutralisation of the Immunoglobulin-Cleaving Activity of Streptococcus equi Subspecies equi IdeE by Blood Sera from Ponies Vaccinated with a Multicomponent Protein Vaccine
Source: Vaccines (Basel). 2025 Oct 17;13(10):1061. doi: 10.3390/vaccines13101061 (PMC12568127; doi:10.3390/vaccines13101061)
Supplement: Supplementary file 1 [file vaccines-13-01061-s001.zip › Suppl_tab_S2_quantif.pdf]

Supplementary table 2: Densitometry of the signals detected in the Western blots and calculation of the IdeE neutralisation titres

Relative quantification of:			**intact IgG1 HC**														
			control reaction														
			-	+													
serum %:					10	2	0.4	10	2	0.4	0.08	0.016	10	2	0.4	0.08	0.016
dil					10	50	250	10	50	250	1250	6250	10	50	250	1250	6250
						d0				d119					d135		
Exp I	group 1	0292	7.988219	1.992494	2.612542	1.772114	1.675772	4.968361	3.234584	2.067363	1.920855	1.682375	5.045748	3.540285	1.755107	1.663658	1.963515
		3161	48.66774	17.21781	17.80618	27.006	14.61191	42.03784	36.69866	13.46147	13.69912	13.90448	39.44947	49.73373	17.80803	15.99123	11.68067
		3756	17.68251	5.895076	11.55523	5.266338	4.830128	13.39573	8.949048	4.002316	3.661061	3.994462	11.50428	11.45746	3.862954	4.122042	4.392408
		9602	48.08689	14.25108	15.76902	15.00309	12.92424	32.2449	12.75077	11.46753	11.7115	11.04546	36.30983	31.62183	10.80458	10.9731	13.23562
						d0				d210					d226		
	group 2	0079	73.0159	23.82781	54.70423	21.09269	17.85143	49.11359	20.56247	17.5861	18.25398	16.94321	65.73103	56.04407	19.76056	15.41845	16.36211
		3038	20.63644	6.181779	3.382068	5.127693	5.150832	14.54172	5.334924	5.024006	4.85047	4.27086	11.95517	13.28937	5.022849	3.538395	4.479393
		3150	23.27759	9.268474	15.72793	7.323417	7.238484	26.28167	24.9297	6.804463	6.472889	7.413148	27.34021	25.80566	8.116843	8.107246	7.809981
		6593	37.04151	13.47331	18.30244	8.965737	6.737975	23.77246	23.49528	7.947068	6.734461	9.400395	26.45421	24.91324	9.189106	6.133758	8.941797
						d0				d392					d408		
Exp I	group 3	0016	108.0535	41.55196	88.10765	50.458	33.78718	82.76354	92.83385	34.46049	24.15433	20.31736	53.63535	69.73055	67.34599	28.26322	25.40821
		2015	322.9312	290.8301	220.6473	112.1656	69.18925	193.5935	268.6258	72.34194	49.8129	62.75484	182.7548	199.2151	211.2301	86.93118	77.60215
		5599	16.40398	5.734396	14.33333	9.190501	7.808128	20.63186	20.50137	9.419239	6.135117	6.663409	18.50875	17.77658	18.32527	8.114883	7.203532
		6187	142.1395	54.61573	123.8086	129.365	61.83531	143.7003	166.3605	119.0608	57.33383	56.48516	134.8976	138.5549	136.1157	156.8872	82.97033
						d0				d224							
Exp I ext	group 3	0016	70.53181	23.81888	37.01596	49.27336	20.26995	99.59085	49.77273	21.62156	15.61763	8.003123					
		2015	59.76185	13.08864	66.13979	44.27479	44.20608	173.6877	84.54107	51.17315	45.79277	24.55188					
		5599	192.963	27.33502	77.84377	41.02273	31.83838	263.9966	71.15286	41.91886	23.93939	18.19714					
		6187			50.63912	23.98574	52.76033	319.737	113.9929	92.00729	90.96937	60.09269					
						d-1				d118					d132		
Exp IV	placebo	1819	46.59412	14.44499	18.73619	17.24724	12.14207	22.481	16.64892	9.582413	7.324127	7.254087	18.86854	14.01392	8.410959	7.620857	8.054795
		2084	8.785014	3.132807	5.257335	3.739346	2.873908	4.7469	4.896379	3.030102	2.007249	1.871161	3.699821	3.712525	2.558315	2.258365	2.183244
		2156	37.10613	16.72396	23.53508	18.73197	15.2193	24.82044	26.45675	16.27719	13.01848	14.01325	26.55863	24.73377	17.07948	15.54407	16.07506
		2434	70.23131	25.63339	21.65259	16.28123	20.7653	39.4712	30.65575	25.06754	27.82946	27.79919	46.13915	30.69686	35.8525	38.356	39.88638
		2449	84.78462	69.44211	66.95951	82.92875	80.05506	77.36923	91.95466	94.65911	62.36518	75.15223	91.55547	74.50607	91.03482	56.85749	94.13198
		2943	83.85467	70.12492	51.22696	53.141	54.14286	47.17873	49.15152	41.86704	37.38343	37.12925	44.8726	50.62214	49.22511	36.6308	33.69759
		8448	66.29105	60.13006	55.07356	54.90192	39.54744	47.15618	47.03305	36.63913	36.54584	40.57569	51.43923	49.07143	36.3742	37.65618	35.73294
		8679	153.9465	127.7347	109.7347	98.72575	87.7971	85.46154	74.87848	75.79041	75.28651	82.72018	106.7592	108.0457	102.1739	108.3222	117.214
	vaccinated	1235	14.14665	2.557519	2.639994	2.445571	2.827761	8.011805	8.987403	3.490097	3.581287	4.186658	8.74489	9.990572	3.464269	2.979005	3.111393
		2726	39.34422	9.869143	8.444915	6.950897	7.171486	22.19865	22.45862	7.274676	6.109671	5.982303	16.72832	19.68171	28.4489	7.920239	7.550349
		2997	91.46352	26.52516	24.48333	20.25472	18.90346	64.12642	60.16981	20.42956	18.94843	19.36824	61.84214	58.41195	18.59057	17.24308	17.83082
		3121	63.2978	18.07573	18.59345	13.38265	10.9275	30.31069	13.27605	10.61627	9.669441	10.31042	28.22798	16.74839	9.466434	11.52068	23.11896
		4786	53.06	31.45095	25.93488	26.73602	24.78251	34.45402	34.64141	34.98833	29.68339	25.68564	27.61417	28.56134	30.17141	31.05099	25.75384
		4829	15.08386	8.69629	7.921199	7.34769	7.3948	8.042444	8.784593	8.468878	8.255348	6.03977	6.104973	7.337847	7.33944	7.973828	5.105883
		5305	223.9158	138.97	129.445	144.0142	152.8658	127.27	137.5967	148.5958	149.875	111.3125	112.1975	127.63	135.4375	140.0408	96.31833
		6030	90.03691	57.51705	47.52127	48.01336	47.10053	47.90685	52.74376	59.58981	46.76907	40.15431	37.76872	44.35501	51.16837	52.42601	40.80703

			intact/cleaved																	
			-	+																
serum %:					10	2	0.4		10	2	0.4	0.08	0.016		10	2	0.4	0.08	0.016	
dilution					10	50	250	titre	10	50	250	1250	6250	titre	10	50	250	1250	6250	titre
						d0					d119						d135			
Exp I	group 1	0292	7.988219	0.117249	0.164528	0.12094	0.133248	2	2.476592	0.381379	0.154773	0.123705	0.118099	10	1.56168	0.381243	0.12108	0.11028	0.16413	10
		3161	48.66774	0.163011	0.240327	0.438798	0.134782	2	5.005825	1.074443	0.139187	0.14734	0.137533	50	5.549662	5.06452	0.151571	0.123858	0.097146	50
		3756	17.68251	0.160491	0.710227	0.177383	0.172567	2	4.551716	0.684123	0.167653	0.139246	0.157083	10	4.757798	4.934258	0.139939	0.129366	0.146282	50
		9602	48.08689	0.160911	0.176778	0.15477	0.198455	2	3.130873	0.174129	0.147755	0.161059	0.159748	10	3.764136	3.561999	0.180597	0.139774	0.122342	50
						d0					d210						d226			
	group 2	0079	73.0159	0.155603	2.316308	0.16201	0.139719	10	2.217052	0.195638	0.148077	0.131	0.159798	10	6.312405	1.488566	0.15262	0.121479	0.152929	50
		3038	20.63644	0.133741	0.089896	0.170102	0.197948	2	3.937813	0.166752	0.183104	0.167003	0.16865	10	6.422966	7.943295	0.150891	0.188677	0.158312	50
		3150	23.27759	0.141903	0.408653	0.127098	0.116424	2	4.557414	1.784516	0.165963	0.127696	0.118902	50	4.948283	6.698512	0.149029	0.110212	0.098807	50
		6593	37.04151	0.163995	0.351756	0.141672	0.243333	2	3.347954	2.823651	0.238091	0.289784	0.174488	50	5.196342	8.323306	0.296492	0.129595	0.142256	50
						d0					d392						d408			
Exp I	group 3	0016	108.0535	0.195104	6.18833	0.287413	0.17115	10	6.057615	40.48412	0.199923	0.125904	0.108581	50	5.771528	65.99354	39.35455	0.190446	0.180369	50
		2015	322.9312	11.10039	6.64988	0.230859	0.125667	10	6.818223	59.03166	0.176382	0.104778	0.138502	50	5.557583	97.20359	50.11326	0.163105	0.136889	50
		5599	16.40398	0.201939	6.116338	0.292201	0.194511	10	6.83199	28.91512	0.228911	0.135863	0.139506	50	5.033951	36.94687	15.56328	0.175205	0.166644	50
		6187	142.1395	0.249563	7.609611	25.64471	0.228583	50	7.765715	66.70257	0.582589	0.191829	0.165926	50	4.305175	32.41444	60.95813	96.83334	0.355833	1250
						d0					d224									
Exp I ext	group 3	0016	70.53181	1.210005	0.90873	1.192288	0.224281	2	14.09412	0.553461	0.258651	0.198417	0.090874	10						
		2015	59.76185	0.240511	0.481641	0.310048	0.50292	2	5.814318	0.450542	0.303546	0.316267	0.148273	10						
		5599	192.963	0.511717	0.482677	0.376785	0.388177	2	144.1438	0.659536	0.451667	0.330967	0.309622	10						
		6187			1.030092	0.075683	0.342759	10	5.753996	0.293378	0.389284	0.291869	0.266069	10						
						d-1					d118						d132			
Exp IV	placebo	1819	46.59412	0.17986	0.567737	0.351441	0.203349	2	5.203488	0.53705	0.184414	0.123179	0.127955	10	4.774895	0.523584	0.191815	0.156717	0.153254	10
		2084	8.785014	0.2478	1.71874	0.56205	0.322713	10	8.597354	5.368707	0.35711	0.214101	0.198469	50	7.429971	4.380524	0.313839	0.288244	0.270803	50
		2156	37.10613	0.258882	3.516346	0.549134	0.291522	10	4.677979	2.033318	0.313749	0.222738	0.222832	50	4.020945	1.434423	0.306665	0.25447	0.256075	50
		2434	70.23131	0.28645	0.642828	0.361649	0.285631	2	1.026301	0.474574	0.328575	0.312223	0.319766	10	1.06048	0.473931	0.414001	0.402435	0.448479	10
		2449	84.78462	0.530706	0.618262	0.515267	0.579936	2	4.457501	1.499017	0.534712	0.485214	0.539727	50	4.358944	1.1553	0.568369	0.564153	0.571821	50
		2943	83.85467	0.629442	3.268645	1.040983	0.608486	50	4.265236	4.315001	0.663911	0.516707	0.449655	50	4.74583	6.948727	4.998556	0.495669	0.533996	250
		8448	66.29105	0.612746	3.885158	1.14228	0.579853	50	5.165839	2.890643	0.621373	0.67995	0.622139	50	8.097676	3.339064	0.774736	0.717574	0.702695	50
		8679	153.9465	0.596552	0.905522	0.722107	0.710975	2	2.342092	0.626157	0.655078	0.671526	0.601828	10	2.171694	0.717622	0.578946	0.540115	0.660687	10
	vaccinated	1235	14.14665	0.089831	0.156781	0.101694	0.115959	2	3.988995	1.910455	0.115258	0.123425	0.155485	50	3.793581	5.343941	0.137557	0.104991	0.125941	50
		2726	39.34422	0.139696	0.169714	0.121071	0.104223	2	5.181882	4.732602	0.111196	0.08996	0.090523	50	3.2713	26.32099	4.950425	0.115543	0.1167	250
		2997	91.46352	0.190593	0.268441	0.182931	0.15176	2	7.584409	8.197592	0.167762	0.146847	0.154444	50	3.544409	8.760553	0.159253	0.136105	0.133901	50
		3121	63.2978	0.185263	0.273841	0.172667	0.137668	2	4.422907	0.20871	0.140493	0.131873	0.137883	10	5.989459	0.318758	0.133275	0.157387	0.312435	10
		4786	53.06	0.507245	3.445611	0.676625	0.485275	10	5.586922	8.356586	8.131746	0.730322	0.45851	250	5.656515	8.351697	8.688784	1.791312	0.585312	1250
		4829	15.08386	0.512136	2.237736	0.72858	0.570875	10	5.352494	8.051208	5.811237	1.642286	0.616238	1250	6.295162	9.302509	9.993641	4.309197	0.519148	1250
		5305	223.9158	0.610013	5.106983	7.852463	1.774432	250	5.361559	8.85293	9.766404	4.356937	0.636199	1250	6.132687	10.07274	11.39247	5.087153	0.575711	1250
		6030	90.03691	0.612795	2.185018	0.947788	0.660895	10	6.409961	9.144737	6.072099	0.716641	0.549519	250	7.063635	10.30711	9.519618	1.786744	0.640586	1250

cleaved FC

control reaction

-		+													
			10	2	0.4	10	2	0.4	0.08	0.016	10	2	0.4	0.08	0.016
			10	50	250	10	50	250	1250	6250	10	50	250	1250	6250
				d0		d119					d135				
	1	16.99363	15.87905	14.65283	12.5763	2.006128	8.481283	13.35739	15.52774	14.24542	3.230974	9.286176	14.49539	15.0858	11.96318
	1	105.6239	74.09137	61.54546	108.4112	8.397785	34.15598	96.71528	92.97647	101.0992	7.108445	9.820028	117.4901	129.1094	120.2381
	1	36.73145	16.26976	29.68916	27.98993	2.943007	13.08106	23.87262	26.29202	25.42906	2.417984	2.322022	27.60457	31.86336	30.02709
	1	88.56524	89.20223	96.93785	65.1243	10.29901	73.22604	77.61194	72.71583	69.14317	9.646259	8.877551	59.82715	78.50588	108.1855
				d0		d210					d226				
	1	153.1318	23.61699	130.194	127.7665	22.15266	105.105	118.7628	139.343	106.0286	10.41299	37.64971	129.4757	126.9223	106.9918
	1	46.22198	37.62184	30.14476	26.02111	3.692842	31.9932	27.43803	29.04425	25.32379	1.861316	1.67303	33.28793	18.75372	28.29472
	1	65.3155	38.48728	57.62044	62.1737	5.766795	13.97001	41	50.68978	62.34645	5.525192	3.852447	54.46497	73.5607	79.04319
	1	82.15682	52.03163	63.28531	27.69031	7.100593	8.320887	33.37821	23.23962	53.87415	5.090929	2.993191	30.99275	47.33033	62.85724
				d0		d392					d408				
	1	212.9732	14.23771	175.5594	197.4126	13.66273	2.293093	172.3684	191.8469	187.117	9.293093	1.056627	1.711263	148.4051	140.8681
	1	26.2	33.18065	485.8624	550.5742	28.39355	4.550538	410.1441	475.4129	453.0968	32.88387	2.049462	4.215054	532.9763	566.8989
	1	28.39661	2.34345	31.45268	40.14232	3.01989	0.709019	41.14798	45.15672	47.76423	3.676783	0.481139	1.177469	46.31653	43.2272
	1	218.8457	16.27003	5.04451	270.5163	18.50445	2.494065	204.365	298.8798	340.4228	31.33383	4.274481	2.232938	1.620178	233.1721
				d0		d224									
	1	19.68494	40.73375	41.32671	90.37752	7.066129	89.93003	83.59357	78.71097	88.06847					
	1	54.42011	137.3216	142.8	87.89891	29.87242	187.6428	168.5843	144.7913	165.5861					
	1	53.41818	161.2749	108.8758	82.0202	1.831481	107.8832	92.80926	72.33165	58.77205					
			49.15978	316.9234	153.9284	55.56782	388.5526	236.35	311.6787	225.8538					
				d-1		d118					d132				
	1	80.31242	33.00155	49.07578	59.71056	4.320371	31.00066	51.96156	59.45935	56.69244	3.951613	26.76536	43.84932	48.62815	52.55855
	1	12.64248	3.05883	6.653046	8.90546	0.552135	0.912022	8.485064	9.375262	9.427988	0.497959	0.847507	8.151692	7.834917	8.062111
	1	64.60082	6.69305	34.11186	52.20638	5.305805	13.01161	51.87964	58.44759	62.887	6.60507	17.24301	55.6942	61.08422	62.77482
	1	89.48633	33.68331	45.01943	72.6998	38.45968	64.59634	76.29162	89.13327	86.93607	43.50779	64.77073	86.59996	95.30969	88.93698
	1	130.8486	108.3028	160.9433	138.0413	17.35709	61.34332	177.0283	128.5312	139.2413	21.00405	64.49069	160.1684	100.7838	164.6178
	1	111.4082	15.67223	51.04886	88.97959	11.06122	11.39085	63.06122	72.34941	82.57267	9.455164	7.285096	9.847866	73.90167	63.10452
	1	98.1322	14.17537	48.06343	68.20256	9.128465	16.27079	58.96482	53.74787	65.21962	6.352345	14.69616	46.95043	52.47708	50.85128
	1	214.1215	121.1839	136.7191	123.4883	36.48941	119.5842	115.6968	112.1126	137.4482	49.15942	150.5608	176.4827	200.5541	177.4125
	1	28.47045	16.83877	24.04841	24.38591	2.008477	4.704326	30.28078	29.01593	26.92648	2.305181	1.869514	25.18428	28.37379	24.7052
	1	70.64731	49.75972	57.41152	68.80932	4.283898	4.745513	65.42223	67.9155	66.08574	5.113659	0.747757	5.74676	68.54786	64.69865
	1	139.172	91.20566	110.7233	124.5613	8.455031	7.339937	121.7774	129.0352	125.4066	17.4478	6.66761	116.7358	126.6896	133.1642
	1	97.56767	67.89877	77.50537	79.37594	6.853115	63.6101	75.56445	73.32385	74.77658	4.712943	52.5427	71.02927	73.19952	73.99597
	1	62.00348	7.52693	39.51382	51.06902	6.166906	4.145402	4.302683	40.64428	56.01987	4.881835	3.419824	3.472455	17.33422	44.00021
	1	16.98043	3.539827	10.08495	12.95346	1.50256	1.09109	1.457328	5.026741	9.801036	0.969788	0.788803	0.734411	1.850421	9.835116
	1	227.815	25.34667	18.34	86.14917	23.7375	15.5425	15.215	34.39917	174.965	18.295	12.67083	11.88833	27.52833	167.3033
	1	93.86011	21.74868	50.65835	71.26784	7.473814	5.767663	9.813708	65.26151	73.07171	5.346924	4.303339	5.375044	29.34165	63.70264

intact/cleaved

control reaction

-	+													
		10	2	0.4	10	2	0.4	0.08	0.016	10	2	0.4	0.08	0.016
		10	50	250	10	50	250	1250	6250	10	50	250	1250	6250
		d0			d119					d135				
7.988219	0.117249	0.164528	0.12094	0.133248	2.476592	0.381379	0.154773	0.123705	0.118099	1.56168	0.381243	0.12108	0.11028	0.16413
48.66774	0.163011	0.240327	0.438798	0.134782	5.005825	1.074443	0.139187	0.14734	0.137533	5.549662	5.06452	0.151571	0.123858	0.097146
17.68251	0.160491	0.710227	0.177383	0.172567	4.551716	0.684123	0.167653	0.139246	0.157083	4.757798	4.934258	0.139939	0.129366	0.146282
48.08689	0.160911	0.176778	0.15477	0.198455	3.130873	0.174129	0.147755	0.161059	0.159748	3.764136	3.561999	0.180597	0.139774	0.122342
		d0			d210					d226				
73.0159	0.155603	2.316308	0.16201	0.139719	2.217052	0.195638	0.148077	0.131	0.159798	6.312405	1.488566	0.15262	0.121479	0.152929
20.63644	0.133741	0.089896	0.170102	0.197948	3.937813	0.166752	0.183104	0.167003	0.16865	6.422966	7.943295	0.150891	0.188677	0.158312
23.27759	0.141903	0.408653	0.127098	0.116424	4.557414	1.784516	0.165963	0.127696	0.118902	4.948283	6.698512	0.149029	0.110212	0.098807
37.04151	0.163995	0.351756	0.141672	0.243333	3.347954	2.823651	0.238091	0.289784	0.174488	5.196342	8.323306	0.296492	0.129595	0.142256
		d0			d392					d408				
108.0535	0.195104	6.18833	0.287413	0.17115	6.057615	40.48412	0.199923	0.125904	0.108581	5.771528	65.99354	39.35455	0.190446	0.180369
322.9312	11.10039	6.64988	0.230859	0.125667	6.818223	59.03166	0.176382	0.104778	0.138502	5.557583	97.20359	50.11326	0.163105	0.136889
16.40398	0.201939	6.116338	0.292201	0.194511	6.83199	28.91512	0.228911	0.135863	0.139506	5.033951	36.94687	15.56328	0.175205	0.166644
142.1395	0.249563	7.609611	25.64471	0.228583	7.765715	66.70257	0.582589	0.191829	0.165926	4.305175	32.41444	60.95813	96.83334	0.355833
		d0			d224									
70.53181	1.210005	0.90873	1.192288	0.224281	14.09412	0.553461	0.258651	0.198417	0.090874					
59.76185	0.240511	0.481641	0.310048	0.50292	5.814318	0.450542	0.303546	0.316267	0.148273					
192.963	0.511717	0.482677	0.376785	0.388177	144.1438	0.659536	0.451667	0.330967	0.309622					
		1.030092	0.075683	0.342759	5.753996	0.293378	0.389284	0.291869	0.266069					
		d-1			d118					d132				
46.59412	0.17986	0.567737	0.351441	0.203349	5.203488	0.53705	0.184414	0.123179	0.127955	4.774895	0.523584	0.191815	0.156717	0.153254
8.785014	0.2478	1.71874	0.56205	0.322713	8.597354	5.368707	0.35711	0.214101	0.198469	7.429971	4.380524	0.313839	0.288244	0.270803
37.10613	0.258882	3.516346	0.549134	0.291522	4.677979	2.033318	0.313749	0.222738	0.222832	4.020945	1.434423	0.306665	0.25447	0.256075
70.23131	0.28645	0.642828	0.361649	0.285631	1.026301	0.474574	0.328575	0.312223	0.319766	1.06048	0.473931	0.414001	0.402435	0.448479
84.78462	0.530706	0.618262	0.515267	0.579936	4.457501	1.499017	0.534712	0.485214	0.539727	4.358944	1.1553	0.568369	0.564153	0.571821
83.85467	0.629442	3.268645	1.040983	0.608486	4.265236	4.315001	0.663911	0.516707	0.449655	4.74583	6.948727	4.998556	0.495669	0.533996
66.29105	0.612746	3.885158	1.14228	0.579853	5.165839	2.890643	0.621373	0.67995	0.622139	8.097676	3.339064	0.774736	0.717574	0.702695
153.9465	0.596552	0.905522	0.722107	0.710975	2.342092	0.626157	0.655078	0.671526	0.601828	2.171694	0.717622	0.578946	0.540115	0.660687
14.14665	0.089831	0.156781	0.101694	0.115959	3.988995	1.910455	0.115258	0.123425	0.155485	3.793581	5.343941	0.137557	0.104991	0.125941
39.34422	0.139696	0.169714	0.121071	0.104223	5.181882	4.732602	0.111196	0.08996	0.090523	3.2713	26.32099	4.950425	0.115543	0.1167
91.46352	0.190593	0.268441	0.182931	0.15176	7.584409	8.197592	0.167762	0.146847	0.154444	3.544409	8.760553	0.159253	0.136105	0.133901
63.2978	0.185263	0.273841	0.172667	0.137668	4.422907	0.20871	0.140493	0.131873	0.137883	5.989459	0.318758	0.133275	0.157387	0.312435
53.06	0.507245	3.445611	0.676625	0.485275	5.586922	8.356586	8.131746	0.730322	0.45851	5.656515	8.351697	8.688784	1.791312	0.585312
15.08386	0.512136	2.237736	0.72858	0.570875	5.352494	8.051208	5.811237	1.642286	0.616238	6.295162	9.302509	9.993641	4.309197	0.519148
223.9158	0.610013	5.106983	7.852463	1.774432	5.361559	8.85293	9.766404	4.356937	0.636199	6.132687	10.07274	11.39247	5.087153	0.575711
90.03691	0.612795	2.185018	0.947788	0.660895	6.409961	9.144737	6.072099	0.716641	0.549519	7.063635	10.30711	9.519618	1.786744	0.640586
