# Supplementary figures and images for: Neutralisation of the Immunoglobulin-Cleaving Activity of Streptococcus equi Subspecies equi IdeE by Blood Sera from Ponies Vaccinated with a Multicomponent Protein Vaccine
Source: Vaccines (Basel). 2025 Oct 17;13(10):1061. doi: 10.3390/vaccines13101061 (PMC12568127; doi:10.3390/vaccines13101061)

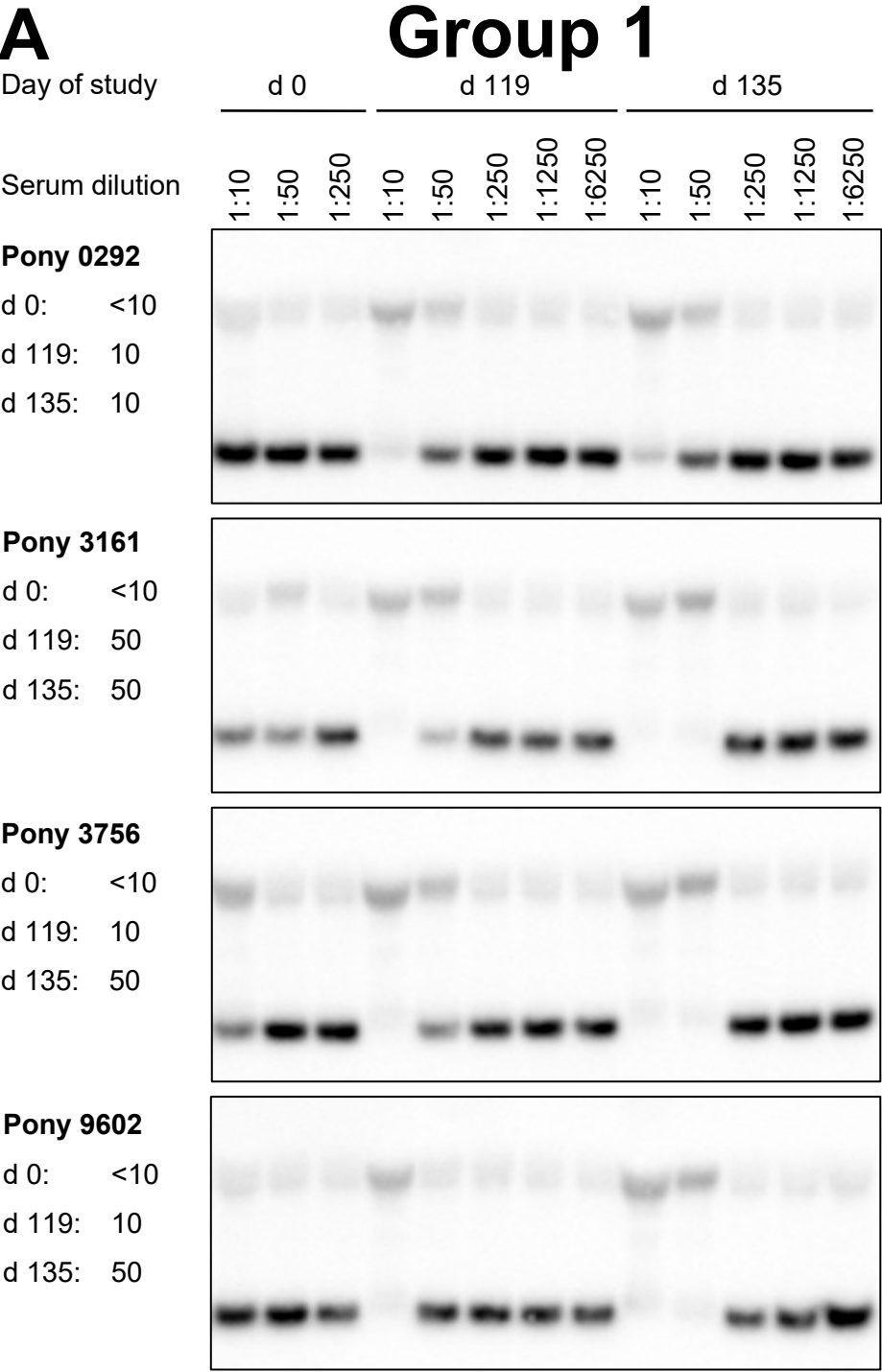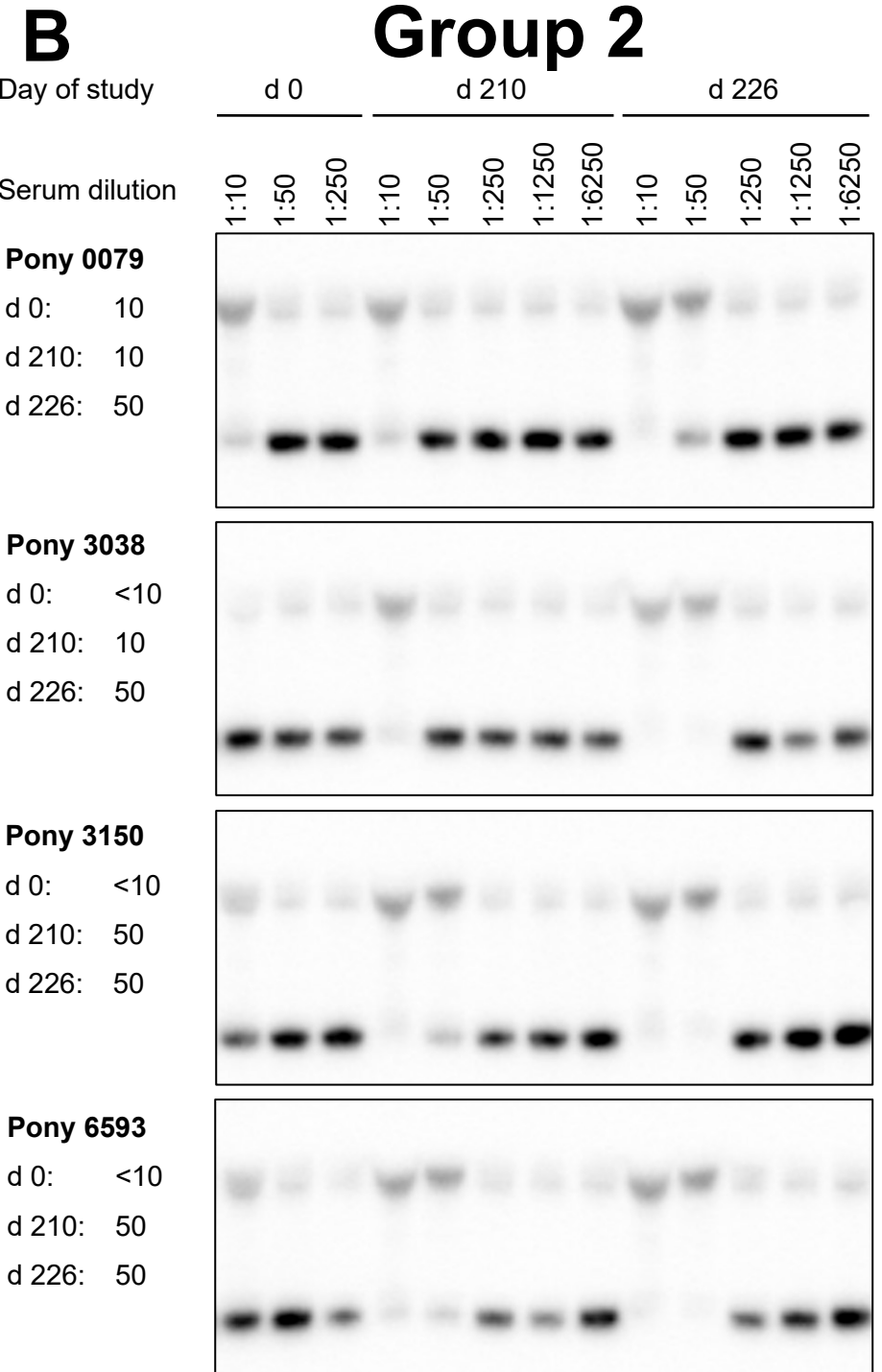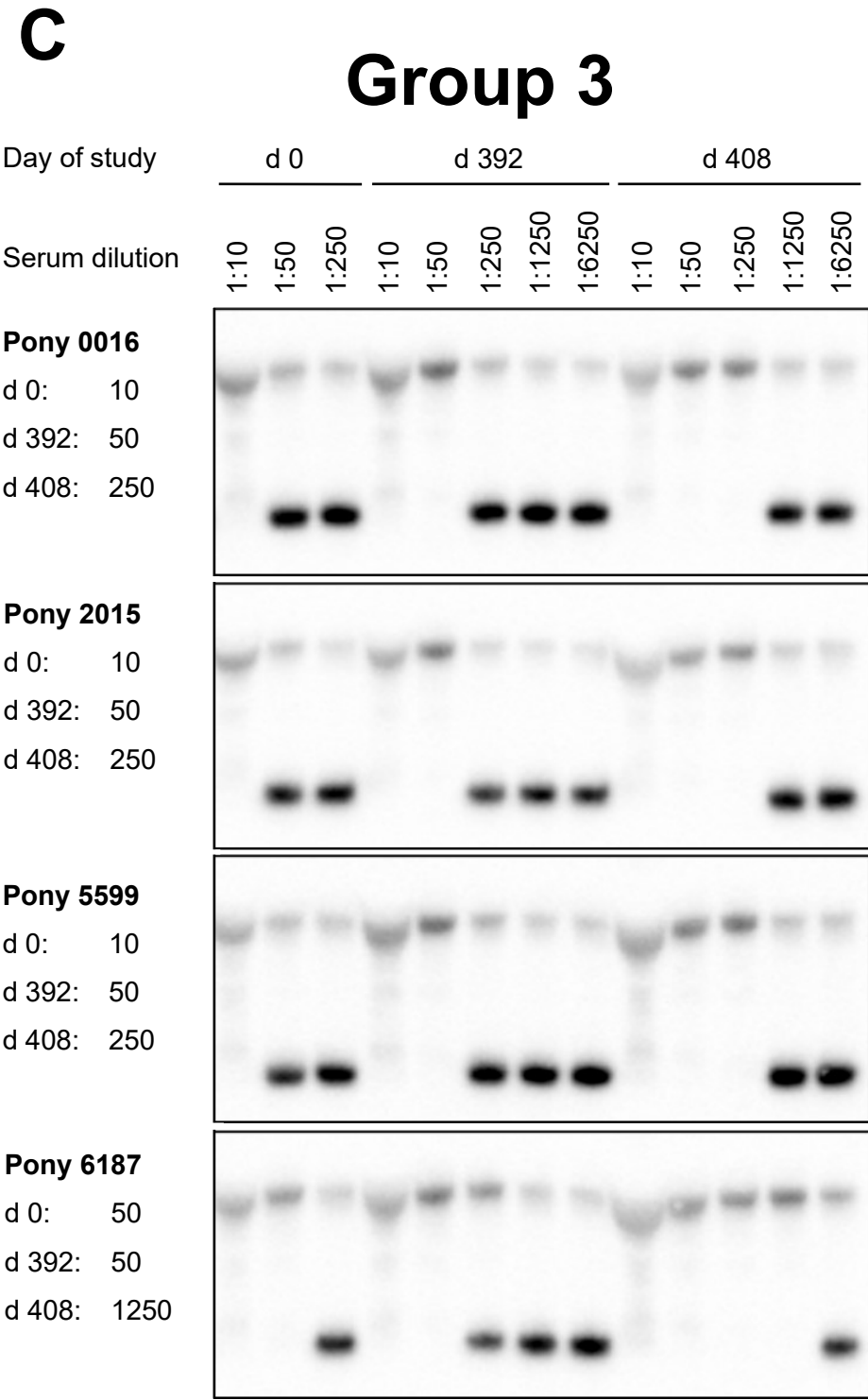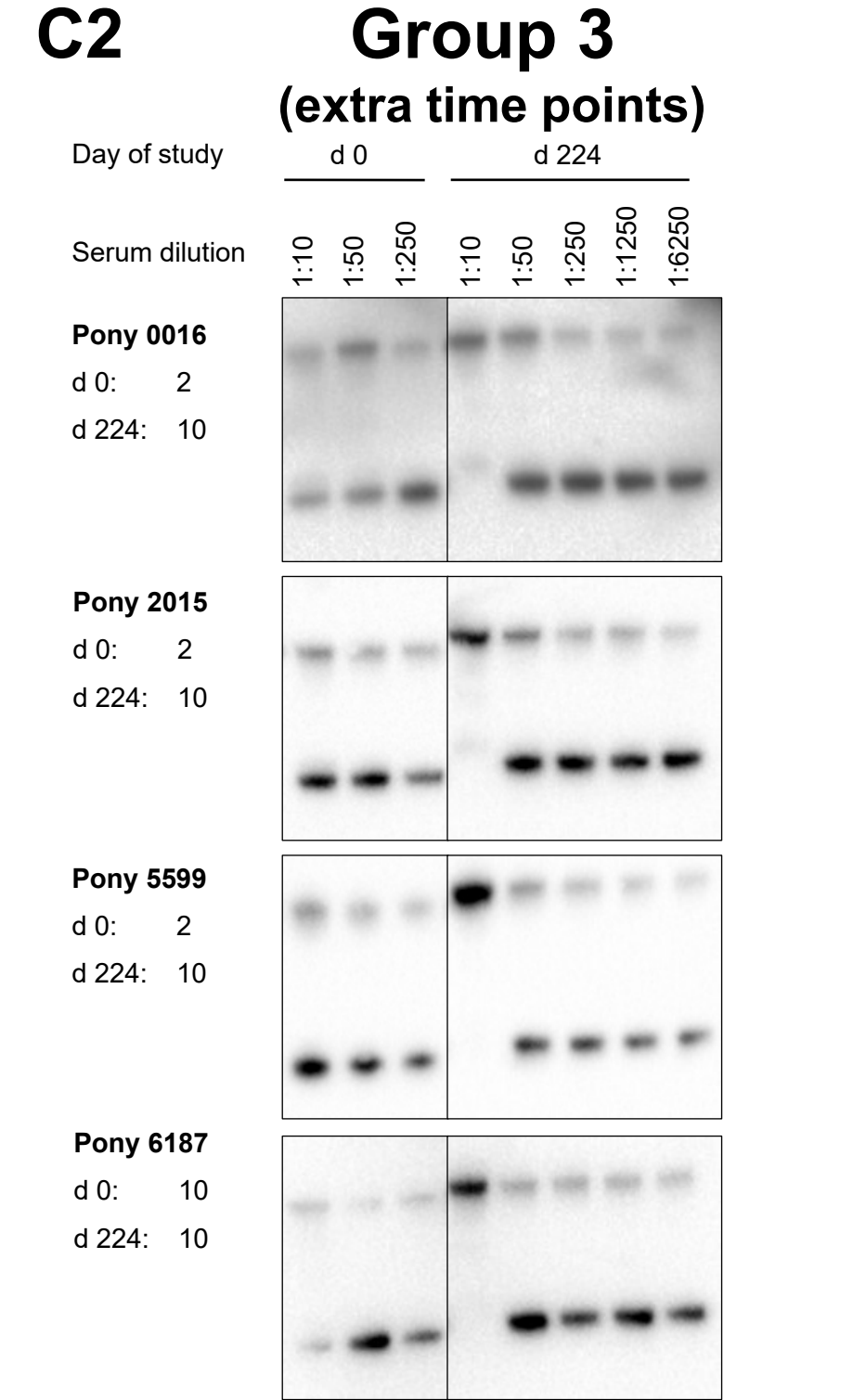

Supplement: Supplementary file 1 [file vaccines-13-01061-s001.zip › Suppl_Fig_S1_ExpIII_wb.pdf]

# Experiment IV

## Placebo group

## Vaccinated group

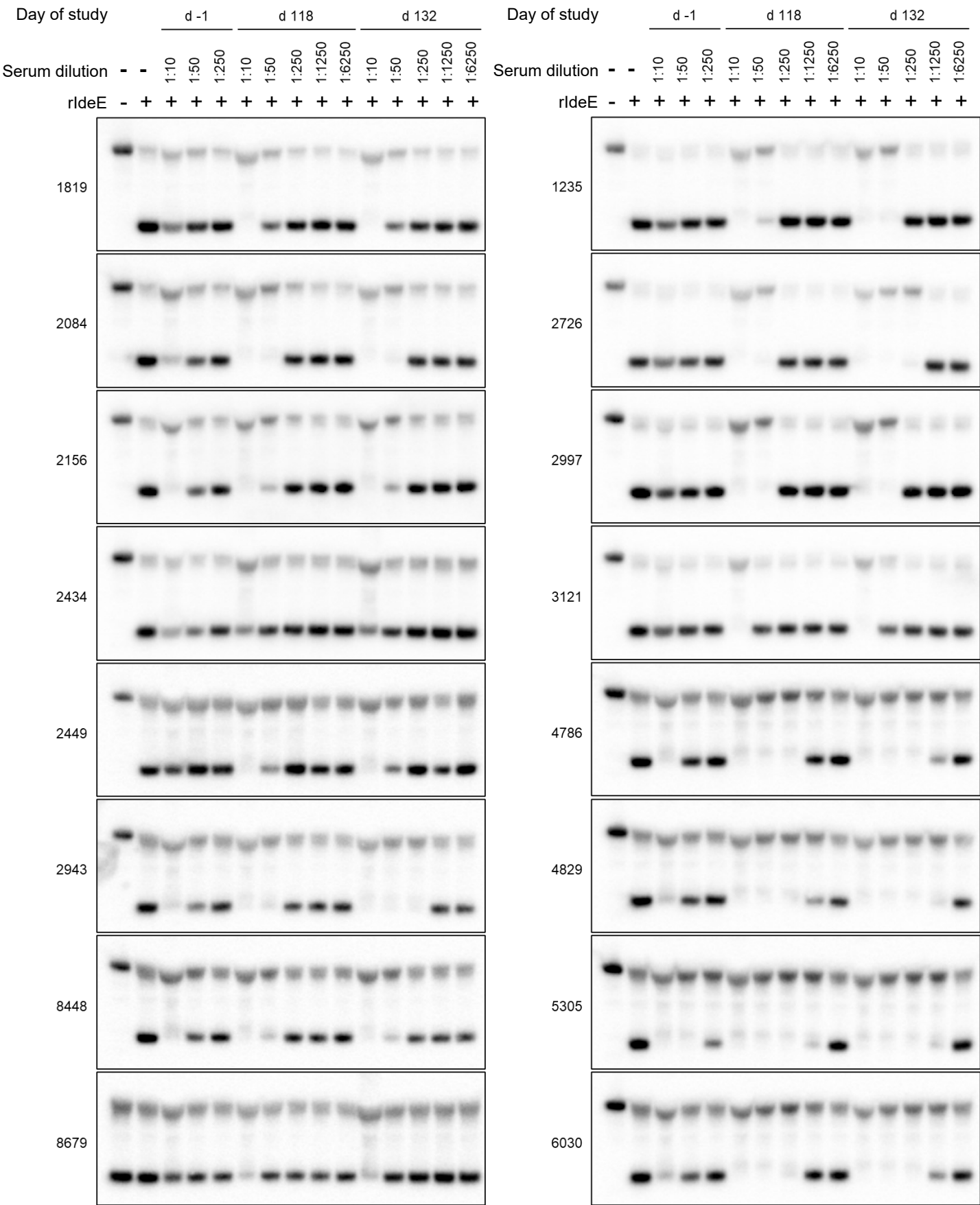

Supplement: Supplementary file 1 [file vaccines-13-01061-s001.zip › Suppl_Fig_S2_ExpIV_wb.pdf]
